# In vivo hyperphosphorylation of tau is associated with synaptic loss and behavioral abnormalities in the absence of tau seeds

**DOI:** 10.1038/s41593-024-01829-7

**Published:** 2024-12-24

**Authors:** Naoto Watamura, Martha S. Foiani, Sumi Bez, Mathieu Bourdenx, Alessia Santambrogio, Claire Frodsham, Elena Camporesi, Gunnar Brinkmalm, Henrik Zetterberg, Saisha Patel, Naoko Kamano, Mika Takahashi, Javier Rueda-Carrasco, Loukia Katsouri, Stephanie Fowler, Emir Turkes, Shoko Hashimoto, Hiroki Sasaguri, Takashi Saito, AFM Saiful Islam, Seico Benner, Toshihiro Endo, Katsuji Kobayashi, Chiho Ishida, Michele Vendruscolo, Masahito Yamada, Karen E. Duff, Takaomi C. Saido

**Affiliations:** 1 Laboratory for Proteolytic Neuroscience, RIKEN Center for Brain Science, Wako, Japan; 2 UK Dementia Research Institute at University College London, London, UK; 3 Department of Chemistry, Centre for Misfolding Diseases, University of Cambridge, Cambridge, UK; 4 Queen Square Institute of Neurology, University College London, London, UK; 5 Department of Psychiatry and Neurochemistry, Institute of Neuroscience and Physiology,The Sahlgrenska Academy at the University of Gothenburg, Mölndal, Sweden; 6 Sainsbury Wellcome Centre for Neural Circuits and Behaviour, University College London, London, UK; 7 Nuffield Department of Medicine, Oxford-GSK Institute of Molecular and Computational Medicine, Centre for Human Genetics, Oxford, UK; 8 Pioneering Research Division, Medical Innovation Research Center, Shiga University of Medical Science, Otsu, Japan; 9 Dementia Pathophysiology Collaboration Unit, RIKEN Center for Brain Science, Wako, Japan; 10 Department of Neurocognitive Science, Institute of Brain Science, Nagoya City University Graduate School of Medical Sciences, Nagoya, Japan; 11 Department of Neuroscience and Pathobiology, Research Institute of Environmental Medicine, Nagoya University, Nagoya, Japan; 12 Center for Health and Environmental Risk Research, National Institute for Environmental Studies, Tsukuba, Japan; 13 Phenovance LLC, Chiba, Japan; 14 Department of Psychiatry, Awazu Neuropsychiatric Hospital, Ishikawa, Japan; 15 Department of Neurology, NHO Iou National Hospital, Iwade-machi, Japan; 16 Department of Internal Medicine, Division of Neurology, Kudanzaka Hospital, Tokyo, Japan; 17 Department of Neurology and Neurological Science, Tokyo Medical and Dental University, Tokyo, Japan; 18 Kanazawa University, Kanazawa, Japan

**Keywords:** Experimental models of disease, Alzheimer's disease

## Abstract

Tau pathology is a hallmark of several neurodegenerative diseases, including frontotemporal dementia and Alzheimer’s disease. However, the sequence of events and the form of tau that confers toxicity are still unclear, due in large part to the lack of physiological models of tauopathy initiation and progression in which to test hypotheses. We have developed a series of targeted mice expressing frontotemporal-dementia-causing mutations in the humanized *MAPT* gene to investigate the earliest stages of tauopathy. *MAPT*^Int10+3G>A^ and *MAPT*^S305N;Int10+3G>A^ lines show abundant hyperphosphorylated tau in the hippocampus and entorhinal cortex, but they do not develop seed-competent fibrillar structures. Accumulation of hyperphosphorylated tau was accompanied by neurite degeneration, loss of viable synapses and indicators of behavioral abnormalities. Our results demonstrate that neuronal toxicity can occur in the absence of fibrillar, higher-order structures and that tau hyperphosphorylation is probably involved in the earliest etiological events in tauopathies showing isoform ratio imbalance.

## Abstract (web summary)

Mice expressing humanized mutant tau exhibit synaptic loss and behavioral abnormalities in the absence of abnormal tau conformers, suggesting that hyperphosphorylated tau can be pathological, at least in tauopathies caused by tau isoform imbalance.

## Main

The microtubule-associated protein tau is involved in the pathogenesis of several neurodegenerative diseases, including frontotemporal dementia (FTD) spectrum and Alzheimer’s disease (AD). Mutations in the human tau (*MAPT)* gene cause familial forms of FTD^1–3^, which can manifest as progressive supranuclear palsy, corticobasal syndrome, Pick’s disease and globular glial tauopathy. AD is a secondary tauopathy as tau filamentous inclusions are accompanied (or even triggered) by the accumulation of β-amyloid. More than 30 clinically diverse neurodegenerative diseases and syndromes manifest with tauopathy^4^. Although the etiology for most sporadic forms of FTD is unclear, in several of the syndromes the development of tau pathology has been linked to inflammation^5^. Tauopathies are characterized by the relocation of normally soluble tau from axonal compartments to the somatodendritic compartment of neurons, where it accumulates, usually in an abnormally hyperphosphorylated, aggregated, insoluble form. Several tauopathies accumulate tau in glia as well as in neurons. At end stage, in both primary and secondary tauopathies, the accumulation of abnormal tau is associated with neuronal degeneration, synaptic loss and cognitive impairment^6^. The underlying mechanisms by which tau becomes pathological and synaptotoxic remain poorly understood, particularly in the early stages, and effective disease-modifying therapies are currently unavailable^7–9^.

Numerous tau transgenic (Tg) mouse models have been developed to better understand the underlying mechanisms that lead to tau-related diseases^10–14^ (see ref. ^15^ for a comprehensive list). However, the validity of insights resulting from studies with tau Tg mice, especially in relation to the earliest stages of disease pathogenesis, may be affected by transgene-related effects such as overexpression and genome disruption^16^. Matching wild-type (WT) overexpressing transgenics to mutant homologs is difficult as the transgene needs to be integrated at the same locus of the host genome, with an identical copy number, and genome editing approaches that add mutations are not feasible if the transgene is present as several copies. Efforts have been made recently to introduce pathogenic mutations into the mouse *Mapt* gene^17,18^. However, humanizing the entire tau gene is thought to be important for modelling human tauopathies for several reasons, including recapitulating splice-form ratios, and for modelling tau propagation, as pathological tau has been shown to propagate faster in the *MAPT* KI brain than in WT brain, suggesting a species-defined preference for templating^19^. Several lines of mutant and WT *MAPT* mice that are part of the MODEL-AD consortium have been listed recently^20^. However, no phenotype has been described for any of the lines.

We previously developed a humanized tau knock-in (KI) line, termed *MAPT* KI, in which the mouse *Mapt* gene was replaced with the human *MAPT* gene by homologous recombination^19,21^. *MAPT* KI mice express all six isoforms of human tau under the control of the mouse *Mapt* promoter, resulting in physiologically relevant temporal and spatial expression. *MAPT* KI mice have been used in several studies and are considered to be a relevant model to study tau physiology^22–25^. However, they do not develop overt tau pathology even at an old age^26,27^. *MAPT* alternative splicing results in the generation of six isoforms of tau, three of which contain three microtubule-binding domains (3R), and three contain four microtubule-binding domains (4R). Several mutations in *MAPT* that cause FTD, shift the splice ratio from the usual 1:1 ratio of 3R:4R-tau to an increase of either 3R or 4R-tau. The intronic mutation at position 10 + 3 (Int10+3), located close to the splice-donor site following exon 10, disrupts the stem-loop structure of the pre-mRNA, thereby increasing the inclusion of exon 10, resulting in the overproduction of 4R-tau relative to 3R^3^. Missense mutation S305N, located in the last amino acid of exon 10, also destabilizes the stem-loop structure resulting in the overproduction of 4R-tau^28^. We introduced *MAPT*-S305N and Int10+3 FTD-causing mutations into the *MAPT* KI line. In this study, we describe mouse models of FTD that exhibit hyperphosphorylated tau, synapse loss, neuritic degeneration, and cognitive and behavioral abnormalities, replicating common features observed in patients with *MAPT*-FTLD (frontotemporal lobar degeneration). These mutant mouse models will provide unparalleled insight into the mechanistic underpinnings of tauopathy at the earliest stage of the disease process, including the temporal ordering of events and cause-and-effect relationships. The models will also serve as a relevant platform for preclinical in vivo screening of therapeutic candidates targeting tauopathy.

## Results

### Generation of isogenic lines of mutant *MAPT* KI mice

**Fig. 1:**
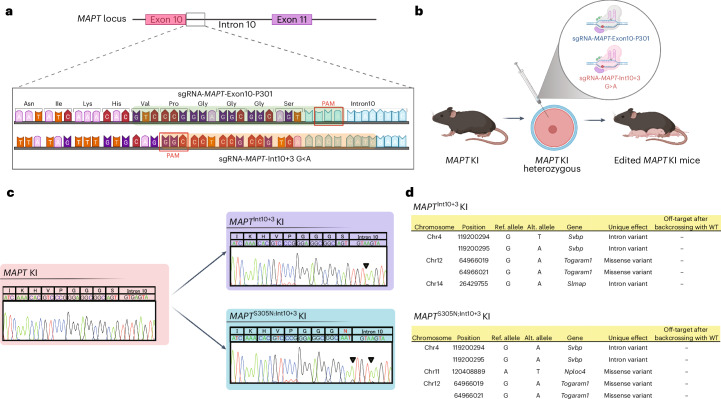
Generation of humanized mutant *MAPT* KI mice with BEs. **a**, Schematic image of BE-mediated genome editing of the *MAPT* gene. **b**, Schematic illustration of BE-mediated genome editing in *MAPT* KI mouse zygotes by microinjection. **c**, Sanger sequencing results determined *MAPT*^Int10+3^ (upper panel) and *MAPT*^S305N;Int10+3^ (lower panel) KI mice. Mutation loci are indicated with arrowheads in black and substituted amino acids highlighted in red. **d**, Regional, genetic and annotated information regarding off-target sites identified in *MAPT*^Int10+3^ and *MAPT*^S305N;Int10+3^ KI mice. Alt., alternative; chr, chromosome; ref., reference. Schematic in **a** created using BioRender.com.

**Extended Data Fig. 1:**
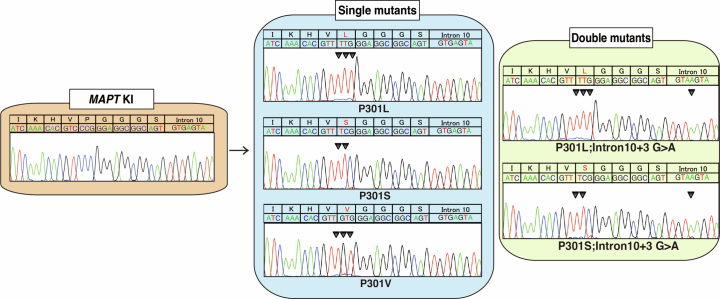
Sanger sequencing of other mutant *MAPT* KI mice. Sanger sequencing results determined *MAPT*^P301L^ KI, *MAPT*^P301S^ KI, *MAPT*^P301V^ KI, *MAPT*^P301L; Int10+3^ KI, and *MAPT*^P301S; Int10+3^ KI mice. Mutation *loci* are indicated by arrowheads in black and the substituted amino acids are highlighted in red.

Third-generation Base Editor (BE3) expressing Cas9 nickase (nCas9) fused with rat APOBEC1 and uracil glycosylase inhibitor (UGI)^29^ was used to create mutant mouse lines. To design the single-guide RNA (sgRNAs) to introduce FTD-associated pathogenic mutations into the *MAPT* KI mice using BE3, we searched for the targetable Cs in the fourth to eighth position of sgRNAs with -NGG (BE) or -NGA (VQR-BE) PAM sequences and found that *MAPT*-P301 (CCG) and *MAPT*-Int10+3 were suitable for the generation of mutant mice carrying *MAPT*-P301L, S (CTG or TCG) with or without the *MAPT*-Int10+3G>A mutation (Fig. 1a)^1,3,30^. The sgRNAs were then microinjected into the cytoplasm of heterozygous *MAPT* KI mouse zygotes together with both BE and VQR-BE (Fig. 1b). Both BE and VQR-BE efficiently converted C to T at the target sites, resulting in the generation of a total of seven lines of tau mutant *MAPT* KI mice (Fig. 1c, Extended Data Fig. 1 and Supplementary Table 1). The fourth position of C at sgRNA-*MAPT*-P301 was converted into T more efficiently compared with the C at the fifth position, which resulted in the substitution of proline (P) to leucine (L) or serine (S) (TTG or TCG) at the targeted position. As a result, mutant *MAPT* KI mice harboring *MAPT*-P301L (47.6%) were obtained more frequently than those carrying *MAPT*-P301S (11.4%). Similarly, double mutant lines harboring *MAPT*-P301L-Int10+3G>A (5.4%) were obtained more frequently than those carrying *MAPT*-P301S-Int10+3G>A (2.4%) (Supplementary Table 1). All mutant *MAPT* KI mice harboring P301 mutations contained the conversion of the third C to T as a silent mutation *MAPT*-V300V (GTC to GTT) (Extended Data Fig. 1). *MAPT* KI mice with the single *MAPT*-Int10+3G>A mutation were also obtained (21.7%). We also generated mutant *MAPT* KI mice harboring *MAPT*-P301V (0.6%) and *MAPT*-S305N-Int10+3G>A (0.6%) mutations due to the inaccurate editing and relatively low specificity of the sgRNA (Fig. 1c and Supplementary Table 1).

Although several mutant *MAPT* mouse lines were generated, for this study we have focused our attention on the splice-shifting mutations that create more 4R-tau. Mice expressing the single mutation at Int10+3 were chosen as they genetically reproduce human FTD^3^. The double mutation line *MAPT*-S305N-Int10+3 was chosen as it has higher levels of 4R-tau than Int10+3 alone. Although the S305N mutation is a missense, exonic mutation, its primary effect is thought to be at the RNA level and, unlike other exonic mutations, it does not reduce microtubule assembly^31^.

### Assessment of off-target sites in mutant *MAPT* KI mice

**Extended Data Fig. 2:**
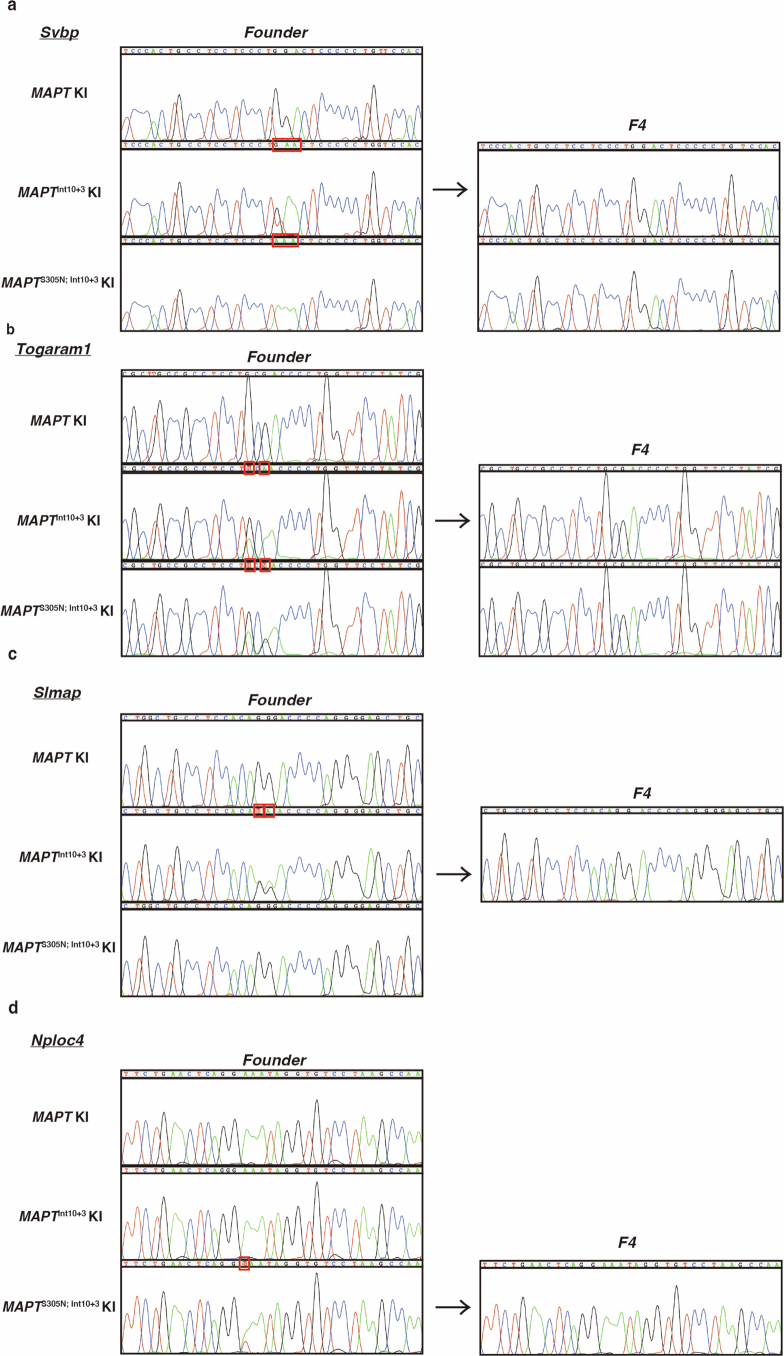
Removal of off-target mutations in *MAPT*^Int10+3^ KI and *MAPT*^S305N; Int10+3^ KI mice by backcrossing with C57B6/J mice. **a-d**, Off-target mutations identified in founders (highlighted in red boxes) were removed by backcrossing with C57B6/J mice more than four times.

The COSMID system was used to search for potential off-target sites of BEs in the genome of *MAPT* KI mice harboring *MAPT*-Int10+3G>A and *MAPT*-S305N-Int10+3G>A mutations (termed *MAPT*^Int10+3^ and *MAPT*^S305N;Int10+3^ KI mice, respectively)^32^; 65 potential sites were identified (Supplementary Table 2) and whole-genome sequencing showed actual off-target mutations (Fig. 1d and Supplementary Table 2). All sites were removed by backcrossing with WT mice (Extended Data Fig. 2a–c). Examination of single-nucleotide variant data and short insertion and deletion analysis data within the coding region of chromosome 11 (where the *Mapt* gene is located) identified a single off-target mutation that corresponded to the missense variant on the *Nploc4* gene in the founder *MAPT*-S305N-Int10+3G>A mouse (Fig. 1d). This off-target mutation was also removed by backcrossing with WT mice. As the mutation could be removed by backcrossing, we assume it was introduced on the allele not containing the expected *MAPT* mutation, or because of inefficient germline transmission (Extended Data Fig. 2d).

### Int10+3 and S305N-Int10+3 lines splice towards 4R-tau

**Fig. 2:**
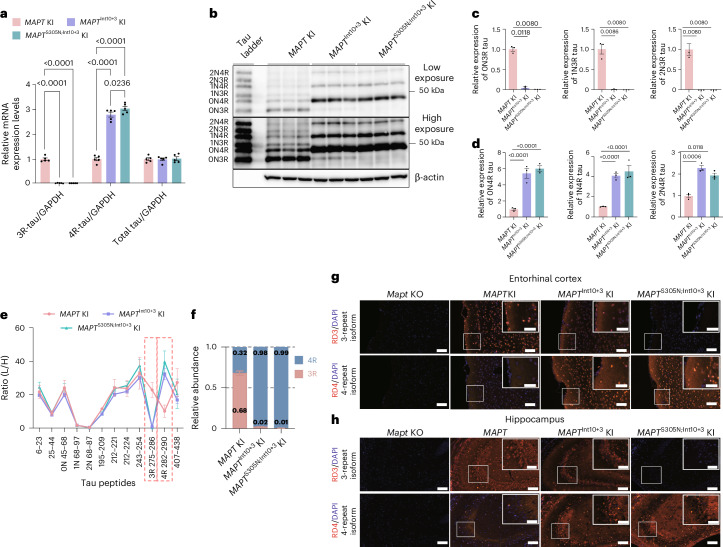
Shift from 3R- to 4R-tau expression induced by Int10+3G>A and S305N-Int10+3G>A mutations in *MAPT* KI mice. **a**, Real-time PCR results of 3R-tau, 4R-tau and total tau levels in *MAPT* KI, *MAPT*^Int10+3^ KI and *MAPT*^S305N;Int10+3^ KI mice (*n* = 5 for each group; *n* = 3 female and *n* = 2 male) using specific primers validated in *Mapt* KO mice. **b**, Immunoblotting of tau detected by tau13 antibody in *MAPT* KI, *MAPT*^Int10+3^ KI and *MAPT*^S305N;Int10+3^ KI mice (*n* = 3 for each group, *n* = 1 female, *n* = 2 male) after alkaline phosphatase treatment. These blots were derived from separate membranes, with an equal amount of protein loaded. **c**,**d**, Quantification of 3R (**c**) and 4R (**d**) tau levels as shown in **b**. **e**,**f**, Relative quantification of tau peptides from *MAPT* KI, *MAPT*^Int10+3^ KI and *MAPT*^S305N;Int10+3^ KI mice by LC–MS/MS analysis shown for individual tau peptides (**e**) and 3R and 4R tau levels (**f**). Red outline in **e** describes 3R- and 4R-tau-specific tau peptides, respectively (*n* = 5–9 for each group; for *MAPT* KI: *n* = 6 sex matched; for *MAPT*^Int10+3^ KI: *n* = 5 of which *n* = 3 female and *n* = 2 male; for *MAPT*^S305N;Int10+3^ KI: *n* = 9 of which *n* = 4 female and *n* = 5 male). **g**,**h**, Immunostaining of 3R- and 4R-tau in the entorhinal cortex (**g**) and hippocampal CA3 region (**h**) of 3-month-old *Mapt* KO, *MAPT* KI, *MAPT*^Int10+3^ KI and *MAPT*^S305N;Int10+3^ KI mice. Scale bars, 100 µm (inset, 50 µm), with four biological replicates with similar observations (Extended Data Fig. 3c–e). In **a**, **c** and **d**, data represent mean ± s.e.m. (two-way ANOVA with Tukey’s multiple comparison test). Source data

**Extended Data Fig. 3:**
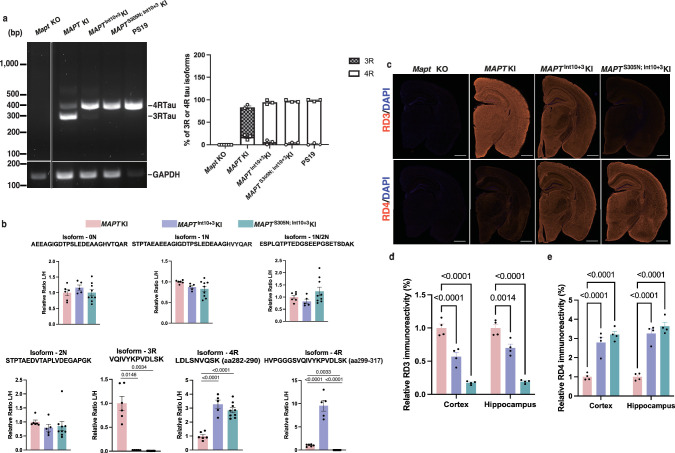
RT-PCR, LC-MS/MS analysis and immunostaining of tau in *MAPT* KI, *MAPT*^Int10+3^ KI and *MAPT*^S305N; Int10+3^ KI mice. **a**, The relative expression of 3 R and 4 R tau were calculated by semi-quantitative RT-PCR using the brain from *Mapt* KO, *MAPT* KI, *MAPT*^Int10+3^ KI, and *MAPT*^S305N; Int10+3^ KI, and PS19 (n = 3 for each group). **b**, Amount of tau peptides based on the isoform resulting from alternative splicing by LC-MS/MS analysis in the brains of 12-15 month-old *MAPT* KI (n = 6; sex-matched), *MAPT*^Int10+3^ KI (n = 5; n = 3 female and n = 2 males), and *MAPT*^S305N; Int10+3^ KI mice(n = 9; n = 4 females and n = 5 male). 4R-tau (299-317 aa) in *MAPT*^S305N; Int10+3^ KI line was not detected because serine (S) at position 305 was converted to asparagine (N). **c-e**, Immunostaining of 3 R and 4 R tau in the brains of 3-month-old *MAPT* KO, *MAPT* KI, *MAPT*^Int10+3^ KI, and *MAPT*^S305N; Int10+3^ KI mice (n = 4 for each group, n = 2 females and n = 2 males). Scale bar represents 1 mm. Data in (**b**), (**d**), and (**e**) represent the mean ± SEM. (two-way ANOVA followed by Tukey’s multiple comparison test). Source data

Real-time PCR (RT-PCR) and semiquantitative RT-PCR analysis showed that expression of 3R-tau was not detected in *MAPT*^S305N;Int10+3^ KI mice but was minimally expressed in the *MAPT*^Int10+3^ KI line compared with *MAPT* KI. Expression of 4R-tau was highest in *MAPT*^S305N;Int10+3^ KI mice followed by *MAPT*^Int10+3^ KI. Total tau levels were unchanged between the lines (Fig. 2a and Extended Data Fig. 3a). Western blot analysis showed low levels of 3R-tau in *MAPT*^Int10+3^ KI mice and undetectable levels of 3R-tau in *MAPT*^S305N;Int10+3^ mice, even at higher exposure. Both mutant lines had relatively high levels of the 0N4R isoform, but both the 1N4R and 2N4R forms were also present (Fig. 2b–d). These data were confirmed by mass spectrometry, showing that the peptide accounting for 3R-tau (amino acids (aa) 275–286) was expressed at low levels in both *MAPT*^Int10+3^ and *MAPT*^S305N;Int10+3^ KI mice (<1%), while 4R-tau (aa 282–290) was enriched in both *MAPT*^Int10+3^ (98%) and *MAPT*^S305N;Int10+3^ KI mice (99%) (Fig. 2e,f and Extended Data Fig. 3b). Finally, immunostaining with antibodies specifically recognizing 3R- or 4R-tau showed that 3R-tau immunoreactivity was absent in *MAPT*^S305N;Int10+3^ KI mice and sparse in *MAPT*^Int10+3^ KI mice. In contrast, 4R-tau immunoreactivity was relatively high in the entorhinal cortex and hippocampal CA3 region of both *MAPT*^Int10+3^ and *MAPT*^S305N;Int10+3^ KI mice (Fig. 2g,h and Extended Data Fig. 3c–e).

### Tau in mutant KI mice is hyperphosphorylated

**Fig. 3:**
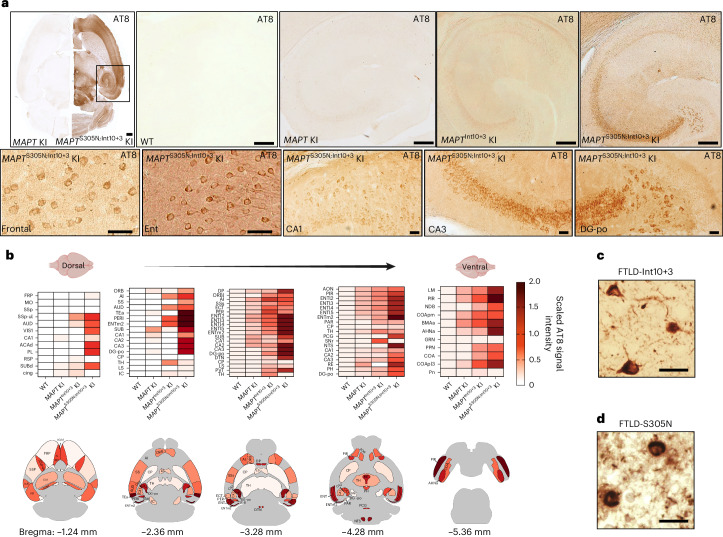
Map of pathological tau detected by AT8 antibody staining in *MAPT*^Int10+3^ and *MAPT*^S305N;Int10+3^ mice. **a**, Immunostaining of phosphorylated tau detected by AT8 antibody in the brains of 15-month-old WT, *MAPT* KI, *MAPT*^Int10+3^ KI and *MAPT*^S305N;Int10+3^ KI mice; *n* = 6 mice were used for each strain, *n* = 3 male and *n* = 3 female. Scale bars, 200 µm (upper panel), 50 µm (lower panel). **b**, Heatmap showing AT8 signal intensity from **a** in each region. **c**, Immunostaining of phosphorylated tau detected by AT8 antibody in the frontal cortex of patients with FTLD with the Int10+3 mutation on the *MAPT* gene, with three biological replicates with similar observations. Scale bar, 50 µm. **d**, Immunostaining of phosphorylated tau detected by AT8 antibody in the frontal cortex of patients with FTLD with the S305N mutation on the *MAPT* gene, with at least three technical replicates with similar observations. Scale bar, 50 µm. ACAd, anterior cingulate area, dorsal part; AHNa, anterior hypothalamic nucleus, anterior part; AI, agranular insular area; AON, anterior olfactory nucleus; AUD, auditory area; BMAa, basomedial amygdalar nucleus, anterior part; CA1, hippocampal CA1; cing, cingulum bundle; CA2, hippocampal CA2; CA3, hippocampal CA3; COA, cortical amygdalar area; COApl3, cortical amygdalar area, posterior part, lateral zone, layer3; COApm, cortical amygdalar area, posterior part, medial zone; CP; caudoputamen; DG-po, dentate gyrus, polymorph layer; DP, dorsal peduncular area; DTN, dorsal tegmental nucleus; ECT, ectorhinal area; ENTI2, entorhinal area, lateral part, layer II; ENTI3, entorhinal area, lateral part, layer3; ENTI4, entorhinal area, lateral part, layer IV; ENTI5, entorhinal area, lateral part, layer5; ENTm2, entorhinal area, medial part, layer2; FRP, frontal pole; GRN, gigantocellular reticular nucleus; IC, inferior colliculus; LM, lateral mammillary nucleus; LS, lateral septal nucleus; MO, somatomotor area; NDB, diagonal band nucleus; NTS, nucleus of the solitary tract; ORB, orbital area; ORB1, orbital area, layer 1; PAR, parasubiculum; PCG, pontine central gray; PER, perirhinal area; PERI, perirhinal area; PH, posterior hypothalamic nucleus; PL, prelimbic area; PPN, pedunculopontine nucleus; PR, pontine nucleus; PVT, paraventricular nucleus of the thalamus (not shown in map); RE, reuniens nucleus; RSP, retrosplenial area; SNr, substantia nigra, reticular part; SS, somatosensory area; SSs; supplementary somatosensory area; SSp, primary somatosensory area; SSP-ul, primary somatosensory area, upper limb; SUB, subiculum; SUBd, subiculum, dorsal part; Tea, temporal association area; TH, thalamus; VIS1, visual area layer 1. Illustrations in **b** created using BioRender.com.

**Fig. 4:**
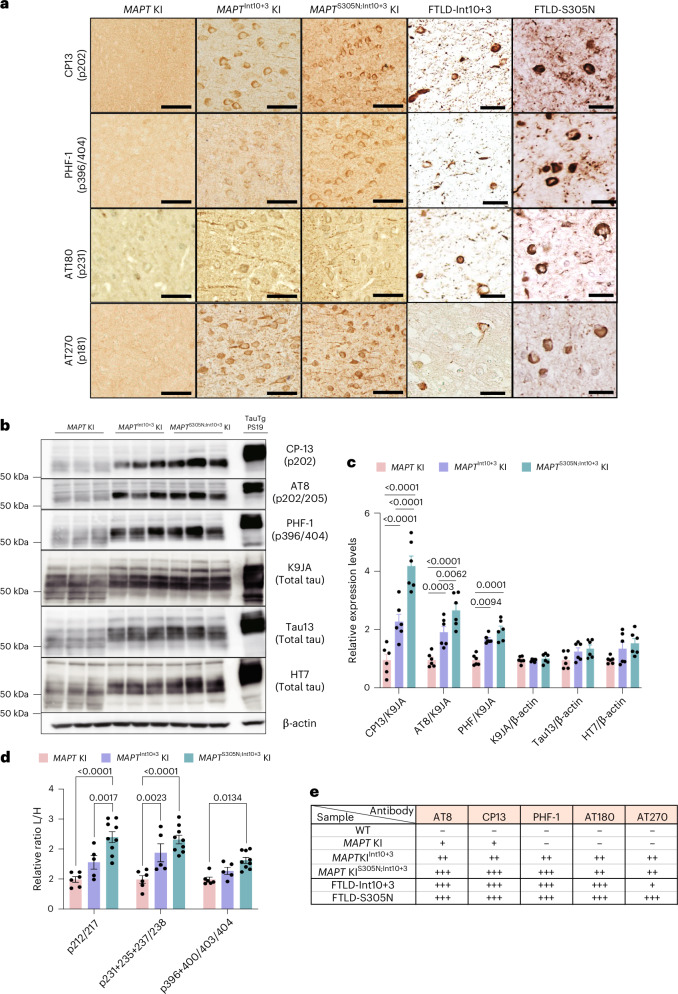
Hyperphosphorylation of tau in *MAPT*^Int10+3^ and *MAPT*^S305N;Int10+3^ mice. **a**, Immunostaining of phosphorylated tau detected by CP13, PHF-1, AT180 and AT270 antibodies in the brains of 15-month-old WT, *MAPT* KI, *MAPT*^Int10+3^ KI and *MAPT*^S305N;Int10+3^ KI mice and patients with FTLD with Int10+3 and S305N mutations on the *MAPT* gene, respectively, with at least three biological replicates with similar observations. Scale bars, 50 µm (mouse data) and 25 µm (data from patients with FTLD). **b**,**c**, Immunoblotting of phosphorylated and total tau detected by CP13, AT8, PHF-1, K9JA, Tau13 and HT7 antibodies in the Tris-soluble fraction of brain lysates from *MAPT* KI, *MAPT*^Int10+3^ KI and *MAPT*^S305N;Int10+3^ KI mice at 12 months of age (*n* = 6 for each group, *n* = 3 female and *n* = 3 males) and in PS19 mice at 9 months of age (**b**) and quantification (**c**). **d**, Relative amount of phospho-tau peptides by LC–MS analysis in the brains of 10- to 18-month-old *MAPT* KI (*n* = 6; sex matched), *MAPT*^Int10+3^ KI (*n* = 5; *n* = 3 female and n = 2 males), and *MAPT*^S305N;Int10+3^ KI mice (*n* = 9; *n* = 4 females and *n* = 5 males). **e**, Summary of histological visual quantification analyses of tau phosphorylation detected by several phospho-tau antibodies. In **c** and **d**, the data represent the mean ± s.e.m. (two-way ANOVA Tukey’s multiple comparison test). Source data

**Extended Data Fig. 4:**
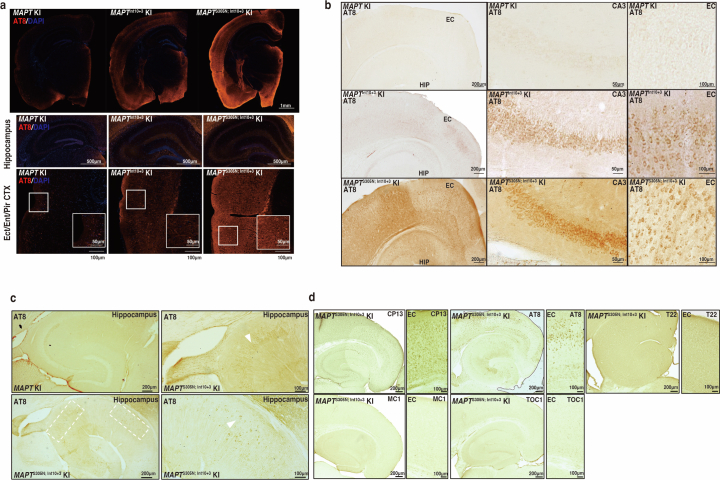
Immunostaining of tau in *MAPT* KI, *MAPT*^Int10+3^ KI and *MAPT*^S305N; Int10+3^ KI mice. **a**, Immunostaining of phosphorylated tau detected by AT8 antibody in the brains of 12-month-old *MAPT* KI, *MAPT*^Int10+3^ KI, and *MAPT*^S305N; Int10+3^ KI mice. Scale bar represents 1 mm in upper panel (hemibrain), 500 µm in the middle panel (hippocampus), 100 µm in the lower panel (piriform/entorhinal (Pir/Ent) cortex) and 50 µm in the insert. **b**, Immunostaining of phosphorylated tau detected by AT8 antibody in the brains of 15-month-old *MAPT* KI, *MAPT*^Int10+3^ KI, and *MAPT*^S305N; Int10+3^ KI mice. Scale bar represents 200 µm in lest panel (hemibrain), 50 µm in the middle panel (CA3), and 100 µm in right panel (EC). **c**, Immunostaining of phosphorylated tau detected by AT8 antibody in the brains of 6-month-old *MAPT* KI, and *MAPT*^S305N; Int10+3^ KI mice. Scale bar represents 200 µm in the left panel and 100 µm in the right panel. Arrowhead indicates AT8 positive signals. **d**, Immunostaining of tau using several anti-tau antibodies (CP13, AT8, T22, MC1, and TOC1) in the brains of 30-month-old *MAPT*^S305N; Int10+3^ KI mice. Scale bar represents 200 µm in the left panel (hemibrain) and 100 µm in the right panel (EC). All experiments were repeated independently at least three times with similar observations.

Positive immunoreactivity was seen for antibodies recognizing phosphorylated tau (phospho-tau) in both mutant lines, but the signal was strongest in *MAPT*^S305N;Int10+3^ KI mice (Fig. 3a and Extended Data Fig. 4a,b). Immunoreactivity was detectable at 6 months of age in *MAPT*^S305N;Int10+3^ KI mice, and it had increased by 15 months of age (Extended Data Fig. 4b,c). There was some variability in signal intensity between mouse lines, but the regions affected were consistent. Phospho-tau immunoreactivity was diffuse in both lines. In addition to the diffuse staining, the somatic compartment of some neurons showed relatively strong immunoreactivity for phospho-tau. Heatmaps were generated based on the intensity and density of somatic staining with the AT8 antibody that recognizes tau phosphorylated at Ser202 and Thr205. The strongest cell body signal was observed in the entorhinal cortex, the subiculum, the polymorphic layer of the dentate gyrus and the pyramidal layer of CA3 and CA2 of the hippocampus (Fig. 3a,b and Extended Data Fig. 4a,b). In both *MAPT*^Int10+3^ and *MAPT*^S305N;Int10+3^ mice, AT8 positive tau accumulation in the entorhinal cortex and parasubiculum was predominantly perinuclear and spread across layer II–IV. Axonal staining was also present in the central entorhinal cortices. In *MAPT*^S305N;Int10+3^ mice, intense signal was also detected in the temporal association cortex, perirhinal, somatosensory and piriform cortices (Fig. 3b). Perinuclear phospho-tau staining was also detected in the nucleus of the solitary tract (NTS; part of the medulla) and the paraventricular nucleus of the thalamus. A similar pattern, albeit less intense and sparser, was observed for *MAPT*^Int10+3^ KI. Phospho-tau immunoreactivity was further assessed using antibodies CP13 (Ser202), PHF-1 (Ser396/Ser404), AT180 (Thr231) and AT270 (Thr181) (Fig. 4a,e). These data were consistent with staining in the frontal cortex of human FTLD-Int10+3 and FTLD-S305N carriers (Figs. 3c,d and 4a). To further analyze the phosphorylation status of tau in *MAPT*^Int10+3^ and *MAPT*^S305N;Int10+3^ KI mice, we performed immunoblotting of the Tris-soluble fraction of brain lysates using several phospho-tau antibodies. Levels of phospho-tau were significantly elevated in both lines assessed by CP13, AT8 and PHF-1 antibodies, whereas the levels of total tau were equivalent across lines (Fig. 4b,c). Finally, similar epitopes were also assessed using a liquid chromatography with tandem mass spectrometry (LC–MS/MS) assay targeting tau peptides. Results consistently showed an increase of phosphorylation at several sites on the proline-rich domain (aa 212, 217, 231, 235 and 238; consistent with AT180 antibody staining results) and the C-terminus (aa 396 and 404; consistent with PHF-1 antibody staining results) of tau in *MAPT*^S305N;Int10+3^ KI mice compared with *MAPT* KI mice (Fig. 4d).

### Tau in mutant KI mice is not conformationally abnormal

**Fig. 5:**
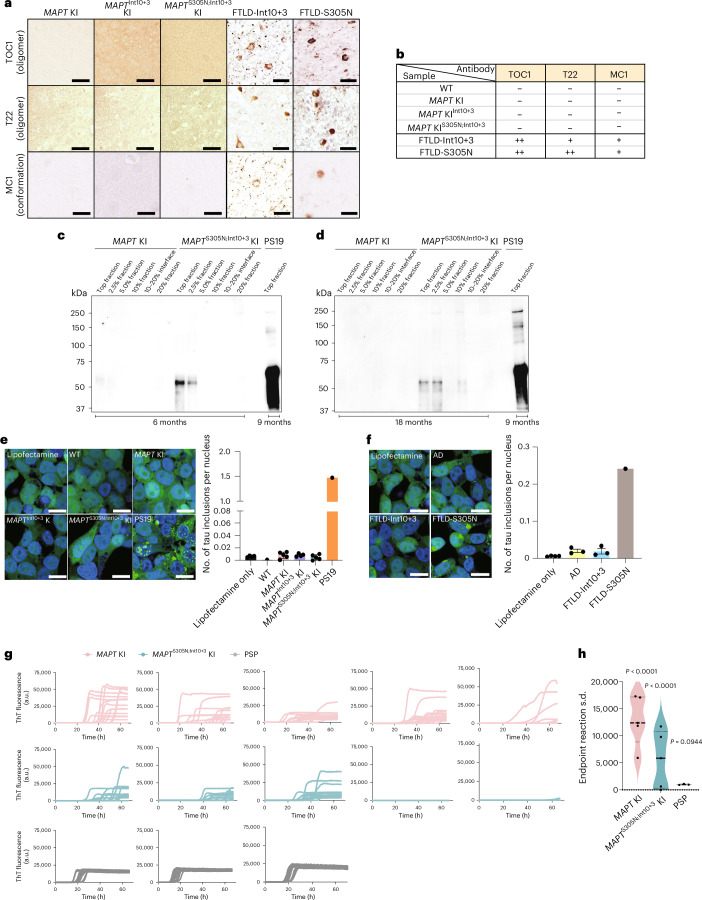
Insolubility and seeding activity of tau in *MAPT*^Int10+3^ and *MAPT*^S305N;Int10+3^ KI mice. **a**, Immunostaining of tau detected by TOC1, T22 and MC1 antibodies in the brains of 15-month-old *MAPT* KI, *MAPT*^Int10+3^ KI and *MAPT*^S305N;Int10+3^ KI mice, and in patients with FTLDs with Int10+3 and S305N mutations in the *MAPT* gene, respectively, with at least three biological replicates with similar observations. Scale bars, 50 µm (mouse data) and 25 µm (data from patients with FTLD). **b**, Summary of histological visual quantification analyses of tau accumulation detected by several tau antibodies. **c**, Immunoblotting of tau detected by CP13 antibody in separated fractions (Top fraction to 20% range) of brain lysates from *MAPT* KI and *MAPT*^S305N;Int10+3^ KI mice at 6 months and from PS19 mice at 9 months. **d**, Immunoblotting of phosphorylated tau detected by CP13 antibody in separated fractions (Top fraction to 20% range) of brain lysates from *MAPT* KI and *MAPT*^S305N;Int10+3^ KI mice at 18 months of age and PS19 mouse at 9 months of age. **e**, Tau seeding activity in brain lysates from WT, *MAPT* KI, *MAPT*^Int10+3^ KI and *MAPT*^S305N;Int10+3^ KI mice at 15 months (*n* = 4–5 for each group, *n* = 2 females and *n* = 2 or 3 males) and PS19 mice at 9 months using S305N biosensor cells. Scale bar, 20 µm. **f**, Tau seeding activity from AD and patients with FTLD with Int10+3 and S305N mutations using S305N biosensor cells (*n* = 3–4 per group, for AD: *n* = 3 females and for Int10+3: *n* = 3 males; *n* = 1 male for FTLD-S305N). Scale bar, 20 µm. **g**, Raw RT-QuIC ThT reactions, 16 replicates, from posterior cortex of mice at 24 months, *MAPT* KI (*n* = 5) and *MAPT*^S305N;Int10+3^ KI (*n* = 5) and positive control PSP motor cortex human brain homogenates (*n* = 3). **h**, Endpoint reaction s.d. for *MAPT* KI (*n* = 5) and *MAPT*^S305N;Int10+3^ KI (*n* = 5) and PSP (*n* = 3). *P* values were calculated as the statistical difference comparing the expected distribution of each group to the observed. In **e** and **f**, data represent mean ± s.e.m. (one-way ANOVA Tukey’s multiple comparison test). In **h**, the box limits show the interquartile range, and the center lines show the median values. Source data

**Extended Data Fig. 5:**
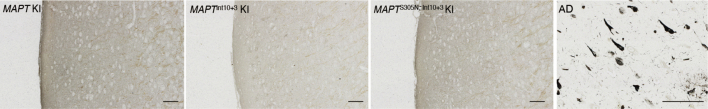
Neurodegenerative analysis in *MAPT* KI, *MAPT*^Int10+3^ KI, *MAPT*^S305N; Int10+3^ KI mice, and AD patient. Gallyas staining in the brains of 15-month-old *MAPT* KI, *MAPT*^Int10+3^ KI, and *MAPT*^S305N; Int10+3^ KI mice (*n* = 3 animals per genotype, n = 2 females and n = 1 male) and AD patient. Scale bar represents 100 µm in mouse samples and 50 µm in the AD patient.

To investigate whether tau in *MAPT*^Int10+3^ and *MAPT*^S305N;Int10+3^ KI mice had undergone a conformational change or oligomerization, we performed immunostaining with antibodies TOC1 (recognizing dimers and oligomers of tau^33^), T22 (recognizing oligomeric tau^34^) and MC1 (recognizing conformationally abnormal tau^35^) (Fig. 5a,b). TOC1 immunoreactivity was only seen in one of the four *MAPT*^S305N;Int10+3^ KI mice tested, and the signal intensity was very low. MC1 and T22 immunoreactivity was not detected in the brains of *MAPT*^Int10+3^ or *MAPT*^S305N;Int10+3^ KI mice even at 30 months of age (Extended Data Fig. 4d). Gallyas silver stain was assessed qualitatively and showed no positive signal (Extended Data Fig. 5).

**Extended Data Fig. 6:**
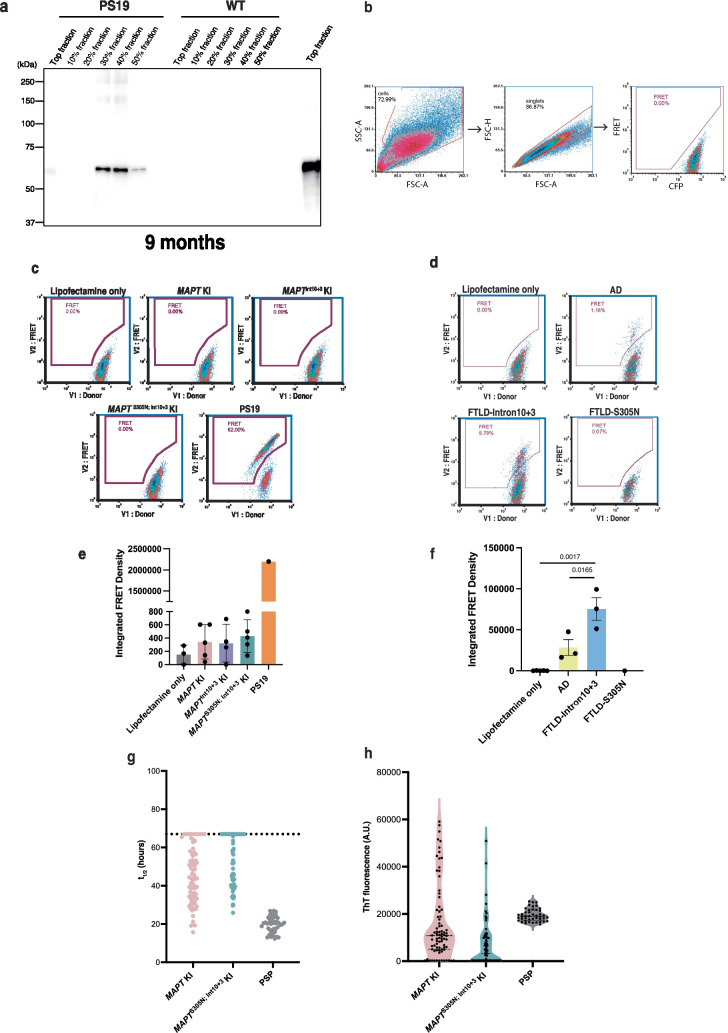
Insolubility of tau in PS19 mice and seeding assays of tau with P301S biosensor cell line. **a**, Immunoblotting of tau detected by CP13 antibody in separated fractions (Top-50% range) of brain lysates from PS19 and WT mice at 9 months of age, with at least three technical replicates with similar observations. **b**, The gating strategy for FRET Flow cytometry. Cell population (left) and singlet/doublet (middle) gates are drawn using standard flow cytometry methodology. A FRET gate (right) is constructed from untreated CFP /YFP cells without YFP single-positive cells. Analysis parameters include: percent FRET positivity, median fluorescence intensity (MFI) of FRET-positive events, and the Integrated FRET Density (Integrated FRET Density = Percent FRET − positive cells × MFI). **c**, FRET *versus* donor (CFP) bivariate plots showing FRET-positive and FRET-negative P301S biosensor cells incubated with brain lysates from *MAPT* KI, *MAPT*^Int10+3^ KI, and *MAPT*^S305N; Int10+3^ KI mice at 15 months and from PS19 mice at 9 months. **d**, FRET *versus* donor (CFP) bivariate plots showing the FRET-positive and FRET-negative P301S biosensor cells incubated with extracts from FFPE-embedded tissue from AD and FTLD patients with Intron10 + 3 and S305N mutations in the *MAPT* gene. **e**, Integrated FRET density from brain lysates from *MAPT* KI (n = 5), *MAPT*^Int10+3^ KI(n = 4) and *MAPT*^S305N; Int10+3^ KI (n = 5) mice at 15 months and PS19 (n = 1) mice at 9 months as a positive control. **f**, Integrated FRET density from AD (n = 3) and FTLD patients with Intron10 + 3 (n = 3) and S305N (n = 1; due to un availability of tissue) mutations on the *MAPT* gene. Data represents the mean ± SEM. (one-way ANOVA with Tukey’s multiple comparison test). **g**, RT-QuIC aggregates half-way curve (t_1/2_) from the combination of the biological replicates from 24 M posterior cortex *MAPT* KI (n = 5) and *MAPT*^S305N; Int10+3^ KI (n = 5) and positive control progressive supranuclear palsy (PSP; n = 3) motor cortex brain homogenates, where the points arranged in a horizontal alignment at 67 h denote reactions where ThT fluorescence levels did not surpass the threshold, suggesting the absence of fibril formation. **h**, RT-QuIC ThT maxima from the combination of biological replicates as in g. Source data

To further investigate whether higher-order conformers of tau had formed in the most affected of the two mutant lines (*MAPT*^S305N;Int10+3^ KI), we fractionated tau species using a sucrose gradient sedimentation assay^36^ followed by sarkosyl extraction and immunoblotting with an antibody recognizing phosphorylated tau (CP13). In 6-month-old mice, sarkosyl-insoluble, phosphorylated tau was present in the top and 2.5% fraction in the mutant but not *MAPT* KI control mice, indicating that the phosphorylated tau in the mutant was mostly in a monomeric state^36^ (Fig. 5c). By 18 months of age, phosphorylated tau species were also identified in the 10% fraction (Fig. 5d). Although the presence of tau in this fraction suggests some higher-order structure, the lack of shift in mobility suggests that tau had not undergone self-aggregation. Sarkosyl-insoluble phosphorylated tau was not observed in brain tissue from *MAPT* KI mice (Fig. 5c,d). In contrast, sarkosyl-insoluble phosphorylated tau was detected in the 30–50% fractions in brain tissue from a tau Tg line with overt pathology (PS19 mouse line), which is consistent with a previous study^36^ (Fig. 5c,d and Extended Data Fig. 6a).

### Tau in *MAPT*^S305N;Int10+3^ KI mice is not seed-competent

To assess whether tau from *MAPT*^Int10+3^ and *MAPT*^S305N;Int10+3^ KI mice had undergone an overt conformational change that enabled prion-like templating (seeding)^37^, we analyzed seeding activity using biosensor cell lines overexpressing either the repeat domain of tau containing the P301S mutation tagged with CFP or YFP (P301S biosensor cell line)^37^ or a biosensor line expressing the S305N mutation tagged with YFP (S305N biosensor cell line). TBS extracts from *MAPT* KI, *MAPT*^Int10+3^ and *MAPT*^S305N;Int10+3^ KI mice did not show any seeding activity in either biosensor cell lines. Extract from PS19 line was used as a positive control (Fig. 5e and Extended Data Fig. 6b,c,e). To confirm that the biosensor cell lines would be capable of detecting seeding activity from the S305N mutation if seeds were present, we added extracts from formalin-fixed, paraffin-embedded (FFPE) sections from brain tissue of patients with FTLD-tau with S305N or Int10+3 *MAPT* mutations, as well as from patients with AD. Tissue from the S305N mutation carrier, but not the FTLD-Int10+3 or AD case, showed seeding activity in the S305N biosensor cells (Fig. 5f). In the P301S biosensor cell line, no activity was detected from human S305N mutation carriers, but both FTLD-Int10+3 and AD brain material seeded (Extended Data Fig. 6b,d,f). To further assess whether tau in *MAPT*^S305N;Int10+3^ KI mice was seed-competent, we performed 4R-tau-specific real-time quaking-induced conversion (RT-QuIC). This method assesses the ability of tau aggregates to act as seeds that can grow by incorporating monomers of tau by seeded polymerization^38,39^. Thioflavin T (ThT) fluorescence RT-QuIC reactions from a positive control sample (extract from a human brain with 4R-tau progressive supranuclear palsy; PSP) showed seeded fibrilization. In PSP, ThT amplitudes all fell within a similar range of ThT fluorescence and with a similar half-life (t_1/2_) (Fig. 5g and Extended Data Fig. 6g,h) indicating that seeds were present and aggregates adopted a specific conformation. In contrast, *MAPT* KI and *MAPT*^S305N;Int10+3^ KI mouse extract showed delayed and variable reactions (Fig. 5g), with no, or highly different, ThT amplitudes and t_1/2._ Some reactions did not surpass the predefined threshold (Extended Data Fig. 6g,h). These data indicate random and spontaneous nucleation and polymerization (Fig. 5h; randomness was assessed as the s.d. at endpoint reaction time, and assessed statistically with *χ*^2^ of s.d., PSP *P* = 0.0944, *MAPT* KI *P* < 0.0001 and *MAPT*^S305N;Int10+3^ KI *P* < 0.0001). This indicates the absence of seed-competent templating ability in the *MAPT* KI and *MAPT*^S305N;Int10+3^ KI brain homogenates.

### *MAPT*^S305N;Int10+3^ KI show synapse loss and early neurodegeneration

**Fig. 6:**
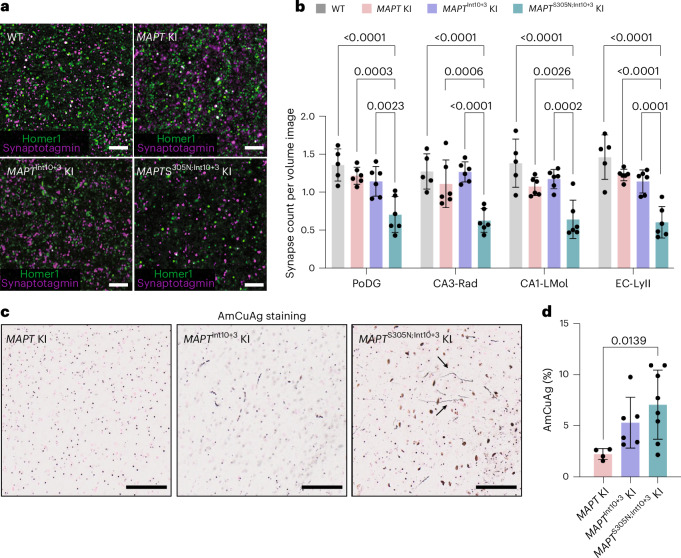
Synaptic loss and neurodegeneration in *MAPT*^S305N;Int10+3^ KI mice. **a**, Representative super-resolution images of Homer1 and Synaptotagmin *puncta* colocalization in the entorhinal cortex layer II (EC-Lyll) of 16-month-old WT, *MAPT* KI, *MAPT*^Int10+3^ and *MAPT*^S305N;Int10+3^ KI mice. Scale bar, 5 µm. **b**, Quantification of Synaptotagmin/Homer1 colocalization density in the polymorph layer of the dentate gyrus (PoDG), CA3 stratum radiatum (CA3-Rad), CA1 lacunosum molecular layer (CA1-Lmol) and EC-LyII regions of 16-month-old WT, *MAPT* KI, *MAPT*^Int10+3^ and *MAPT*^S305N;Int10+3^ KI mice (*n* = 5–6 animals per genotype; *n* = 3 females and *n* = 2 or 3 males). **c**, Amino-cupric silver (AmCuAg) staining in the entorhinal cortices of 15-month-old *MAPT* KI, *MAPT*^Int10+3^ and *MAPT*^S305N;Int10+3^ KI mice. Scale bar, 50 µm. Black arrows, structures resembling neurites. **d**, Quantification of AmCuAg staining (*MAPT* KI, *n* = 4; *MAPT*^Int10+^ KI, *n* = 6; and *MAPT*^S305N;Int10+3^ KI, *n* = 8; sex matched per genotype). In **b**, data represent mean ± s.e.m. (two-way ANOVA with Tukey’s multiple comparison test). In **d**, data represent mean ± s.e.m. (Kruskal–Wallis one-way ANOVA test with Dunn’s multiple comparison test). Source data

**Extended Data Fig. 7:**
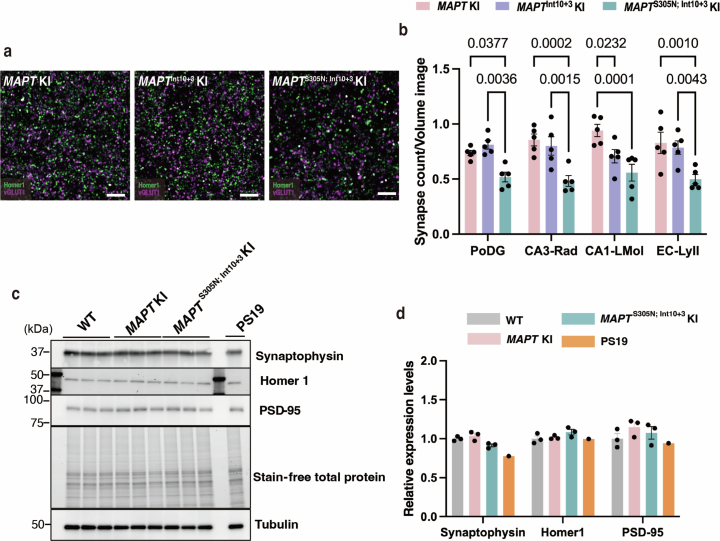
Synaptic loss in *MAPT*^S305N; Int10+3^ KI mice. **a**, Representative super-resolution images of Homer1 and vGLUT1 *puncta* colocalization in the entorhinal cortex layerII (EC-LyII) regions of 16-month-old *MAPT* KI, *MAPT*^Int10+3^, and *MAPT*^S305N; Int10+3^ KI mice. Scale bar represents 5 µm. **b**, Quantification of vGUT1/ Homer1 colocalization density in the polymorph layer of the dentate gyrus (PoDG), CA3 stratum radiatum (CA3-Rad), CA1 lacunosum molecular layer (CA1-Lmol), and entorhinal cortex layerII (EC-LyII) regions of 16-month-old *MAPT* KI, *MAPT*^Int10+3^, and *MAPT*^S305N; Int10+3^KI mice (*n* = 5 animals per genotype). **c,d**, Immunoblotting of synaptophysin, Homer1, PSD-95, and tublin in the tris-soluble fraction of brain lysates from WT, *MAPT* KI and *MAPT*^S305N; Int10+3^ KI mice at 16 months of age (n = 3 for each group, n = 2 female and n = 1 males) and in PS19 (n = 1) mice at 9 months of age. These blots were derived from separate membranes, with an equal amount of protein loaded. In (**b)**, the data represent the mean ± SEM (two-way ANOVA with Tukey’s multiple comparison test). In (**d)**, the data represent the mean ± SEM. Source data

Synapse loss and neurodegeneration are pathological hallmarks of AD and other tauopathies^40–42^. To determine whether the number of viable synapses was affected in *MAPT*^Int10+3^ and *MAPT*^S305N;Int10+3^ KI mice compared with *MAPT* KI mice, we used super-resolution microscopy to quantify the number of puncta labelled with presynaptic (synaptotagmin and vGLUT1) and postsynaptic (Homer1) markers in different hippocampal regions as well as in layer II of the entorhinal cortex. The number of colocalized synaptotagmin/Homer1 immunoreactive puncta was significantly reduced in all regions tested from *MAPT*^S305N;Int10+3^ KI mice (Fig. 6a,b). This result was consistent with a reduction in the number of vGLUT1/Homer1 immunoreactive puncta (Extended Data Fig. 7a,b). A second, less rigorous method (immunoblotting of extracted bulk tissue) was also tested but the protein levels of synaptophysin, homer1, and postsynaptic density protein 95 (PSD-95) were almost equivalent across the lines, suggesting this method was not sensitive enough to detect differences in the tissue extracts (Extended Data Fig. 7c,d).

To assess signs of early neurodegeneration, brain tissues from *MAPT*^Int10+3^ and *MAPT*^S305N;Int10+3^ KI mice were stained with amino-cupric silver^43^, which allows for the detection of neuronal damage. The *MAPT*^S305N;Int10+3^ KI line exhibited significantly increased levels of amino-cupric silver in structures resembling neurites in the entorhinal cortex compared to *MAPT* KI mice (Fig. 6c,d).

### *MAPT*^S305N;Int10+3^ KI display signs of behavioral deficits

**Fig. 7:**
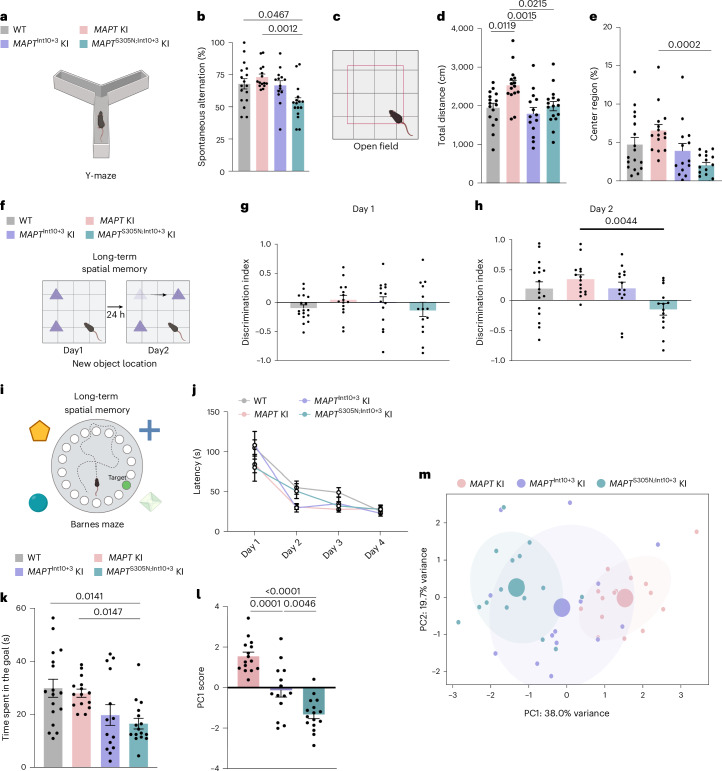
Memory deficits indicated in *MAPT*^S305N;Int10+3^ KI mice. **a**,**b**, Cartoon representation of the test performed (**a**) and percentage of spontaneous alternation of WT (*n* = 17), *MAPT* KI (*n* = 15), *MAPT*^Int10+3^ KI (*n* = 14) and *MAPT*^S305N;Int10+3^ KI (*n* = 16) mice at 15–16 months of age on the Y-maze test (**b**). **c**,**d**, Cartoon representation of the test performed (**c**) and total distance traveled demonstrated by WT (*n* = 16), *MAPT* KI (*n* = 15), *MAPT*^Int10+3^ KI (*n* = 14) and *MAPT*^S305N;Int10+3^ KI (*n* = 16) mice at 15–16 months in the open-field test (**d**). **c**,**e**, Cartoon representation of the test performed (**c**) and percentage of time spent in the center region demonstrated by WT (*n* = 17), *MAPT* KI (*n* = 15), *MAPT*^Int10+3^ KI (*n* = 14) and *MAPT*^S305N;Int10+3^ KI (*n* = 16) mice at 15–16 months in the open-field test (**e**). **f–h**, Cartoon representation of the test performed (**f**) and discrimination index for the NOL test obtained from WT (*n* = 17), *MAPT* KI (*n* = 15), *MAPT*^Int10+3^ KI (*n* = 14) and *MAPT*^S305N;Int10+3^ KI (*n* = 14) mice at 15–16 months at Day1 (**g**) and Day2 (**h**). **i**–**k**, Cartoon representation of the test performed (**i**) and latency from start position to access the escape box on Day1 to Day4 (**j**) and time spent around the target hole at probe test by WT (*n* = 17), *MAPT* KI (*n* = 15), *MAPT*^Int10+3^ KI (*n* = 14) and *MAPT*^S305N;Int10+3^ KI (*n* = 16) mice at 15–16 months of age for the Barnes maze test (**k**). **l**, Values of PC1 scores (individual projection along the main variance axis, PC1) for each experimental group. **m**, PCA was applied on behavioral variables measured using various behavioral tests. Each dot represents a single mouse and larger dots represent the average per genotype. Colored ellipses highlight 1.5 s.d. around the mean per genotype (~87% confidence interval). In **d**, **h** and **l**, data represent mean ± s.e.m. (one-way ANOVA with Tukey’s multiple comparison test). In **b**, **e** and **k**, data represent mean ± s.e.m. (Kruskal–Wallis one-way ANOVA test (Dunn’s multiple comparison test)). In **j**, data represent mean ± s.e.m. (two-way ANOVA with Tukey’s multiple comparison test). Panels **a**, **c**, **f** and **i** were created using BioRender.com. Source data

**Extended Data Fig. 8:**
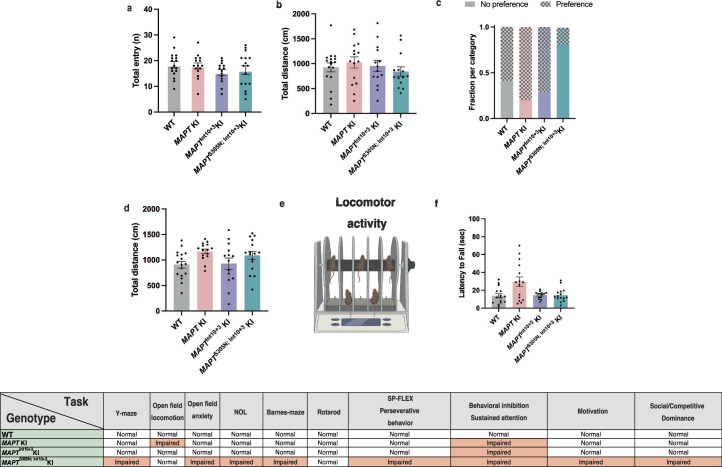
Locomotor performance indicated in *MAPT*^*S305N; Int10+3 G>A*^ KI mice. **a**, Total entry number of WT, *MAPT* KI, *MAPT*^Int10+3^ KI, and *MAPT*^S305N; Int10+3^ KI mice at 15-16 months of age on Y-maze test (WT: n = 17, *MAPT* KI: n = 15, *MAPT*^Int10+3^ KI: n = 14, *MAPT*^S305N; Int10+3^ KI: n = 16). **b**, Total distance of WT, *MAPT* KI, *MAPT*^Int10+3^ KI, and *MAPT*^S305N; Int10+3^ KI mice at 15-16 months of age on the 2^nd^ day of novel object location test (WT: n = 17, *MAPT* KI: n = 15, *MAPT*^Int10+3^ KI: n = 14, *MAPT*^S305N; Int10+3^ KI: n = 14). **c**, Higher failure (defined as <55% preference) in visuospatial memory tests on the novel object location for *MAPT*^S305N; Int10+3^ KI mice compared to WT, *MAPT* KI, *MAPT*^Int10+3^ KI mice at 15-16 months of age (WT: n = 17, *MAPT* KI: n = 15, *MAPT*^Int10+3^ KI: n = 14, *MAPT*^S305N; Int10+3^ KI: n = 14). **d**, Total distance of WT, *MAPT* KI, *MAPT*^Int10+3^ KI, and *MAPT*^S305N; Int10+3^ KI mice at 15-16 months of age on the probe test of Barnes-Maze test (WT: n = 17, *MAPT* KI: n = 15, *MAPT*^Int10+3^ KI: n = 14, *MAPT*^S305N; Int10+3^ KI: n = 16). **e,f**, Latency to fall of WT, *MAPT* KI, *MAPT*^Int10+3^ KI, and *MAPT*^S305N; Int10+3^ KI mice at 15-16 months of age on the Rotarod test (WT: n = 15, *MAPT* KI: n = 15, *MAPT*^Int10+3^ KI: n = 14, *MAPT*^S305N; Int10+3^ KI: n = 15) (created with BioRender.com). **g**, Summary of the behavior tests. In (**b**) and (**d**), the data represent the mean ± SEM (one-way ANOVA with Tukey’s multiple comparison test). In (**c**), the data represent the mean ± SD. In (**f**), the data represent the mean ± SEM (Kruskal-Wallis one-way ANOVA test with Dunn’s multiple comparison test). Source data

To assess whether *MAPT* KI mutants displayed behavioral abnormalities, we conducted a range of tests. Y-maze test results highlighted a significantly reduced spontaneous alternation rate (a sign of spatial working memory deficit) in 15- to 16-month-old *MAPT*^S305N;Int10+3^ KI mice compared with WT and *MAPT* KI mice (Fig. 7a,b). Open-field test results showed that locomotor activity in *MAPT* KI mice was increased significantly compared with that seen in WT and mutant *MAPT* KI mice (Fig. 7c,d). Meanwhile, the percentage of time spent in the center region of the open field was decreased significantly in *MAPT*^S305N;Int10+3^ KI mice compared with *MAPT* KI mice, indicating a heightened level of anxiety in *MAPT*^S305N;Int10+3^ KI mice (Fig. 7c,e). The preference for a new location, assessed by the new object location (NOL) test, was also decreased significantly in *MAPT*^S305N;Int10+3^ KI mice compared with MAPT KI mice (Fig. 7f–h and Extended Data Fig. 8c) indicating impaired long-term spatial memory in these animals. Consistent with these observations, the amount of time spent near the target hole during the probe test of the Barnes maze task was significantly reduced in *MAPT*^S305N;Int10+3^ KI mice compared with WT and *MAPT* KI mice (Fig. 7i,k). No difference was observed among genotypes in the degree of behavioral shaping during the acquisition training (Fig. 7i,j). A significant difference in locomotor activities was not detectable between *MAPT*^S305N;Int10+3^ KI and control mice in the Y-maze, the NOL, the Barnes Maze tests or on the Rotarod test (Extended Data Fig. 8a,b,d–f). All behavioral parameters were then included in a principal component analysis (PCA) to capture broad behavioral changes. We observed a progressive gradient between genotypes, driven mostly by open-field and Y-maze results (Fig. 7l,m).

**Fig. 8:**
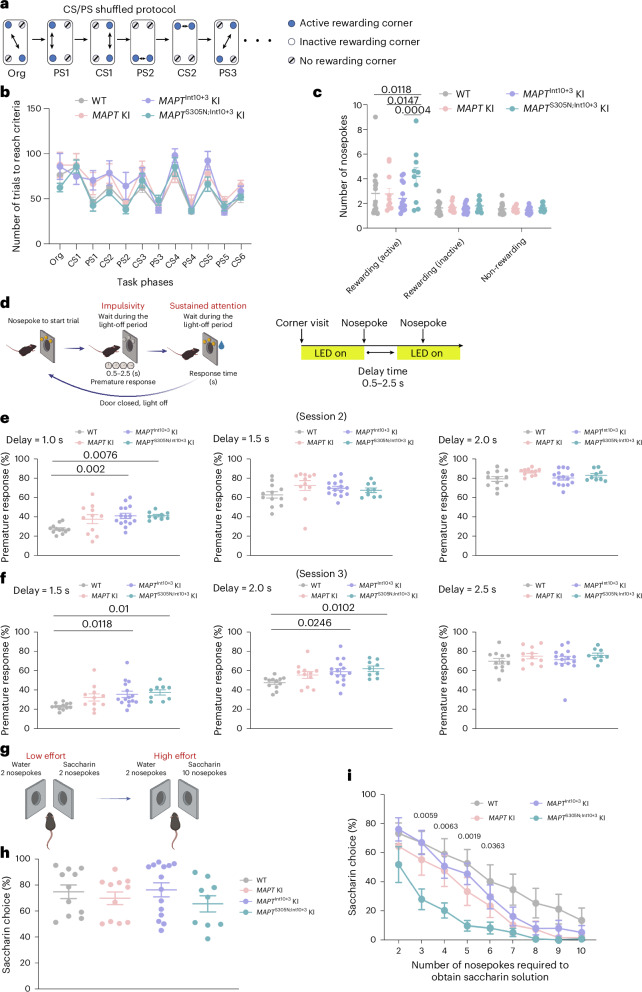
Perseverative, disinhibition behaviors and apathy indicated in *MAPT*^S305N;Int10+3^ KI mice. **a**–**c**, Number of trials to reach criteria and nosepokes of 14- to 16-month-old WT, *MAPT* KI, *MAPT*^Int10+3^ KI and *MAPT*^S305N;Int10+3^ KI mice in the CS/PS-shuffled session of SP-FLEX (WT, *n* = 14; *MAPT* KI, *n* = 11; *MAPT*^Int10+3^ KI, *n* = 15; *MAPT*^S305N;Int10+3^ KI, *n* = 10). **d**–**f**, Percentage of premature responses displayed by 14- to 16-month-old WT, *MAPT* KI, *MAPT*^Int10+3^ KI and *MAPT*^S305N;Int10+3^ KI mice in sessions 2–3 of the behavioral inhibition test (WT, *n* = 12; *MAPT* KI, *n* = 11; *MAPT*^Int10+3^ KI, *n* = 15; *MAPT*^S305N;Int10+3^ KI, *n* = 9). **g**–**i**, Percentages of saccharin choice made by 14- to 16-month-old WT, *MAPT* KI, *MAPT*^Int10+3^ KI and *MAPT*^S305N;Int10+3^ KI mice in the taste preference test and the subsequent effort-based choice test (WT, *n* = 11; *MAPT* KI, *n* = 11; *MAPT*^Int10+3^ KI, *n* = 14; *MAPT*^S305N;Int10+3^ KI, *n* = 9). In **c**, **e**, **f** and **i**, data represent mean ± s.e.m. (two-way ANOVA with Tukey’s multiple comparison test). For **i**, The *P* value shown is the one comparing WT with *MAPT*^S305N;Int10+3^ KI (see source data for results on all comparisons). Illustrations in **d** and **g** created using BioRender.com. Source data

**Extended Data Fig. 9:**
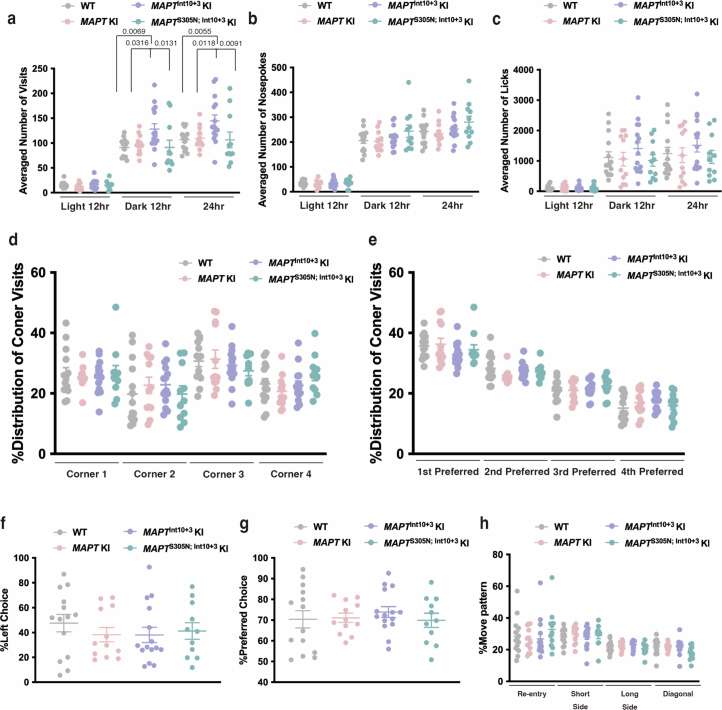
Basal activity parameters calculated from 2 weeks of data in IntelliCage. **a-c**, Average number of visits (**a**), nosepokes (**b**), and licks (**c**) in a day in WT, *MAPT* KI, *MAPT*^Int10+3^ KI and *MAPT*^S305N; Int10+3^ KI mice. **d**, Percentages of corner visit distribution for each corner displayed by WT, *MAPT* KI, *MAPT*^Int10+3^ KI, and *MAPT*^S305N; Int10+3^ KI mice. **e**, Percentages of corner visit distribution in sequence of preferred corners displayed by WT, *MAPT* KI, *MAPT*^Int10+3^ KI, and *MAPT*^S305N; Int10+3^ KI mice. **f**, Percentages of left bottle choice exhibited by WT, *MAPT* KI, *MAPT*^Int10+3^ KI, and *MAPT*^S305N; Int10+3^ KI mice. **g**, Percentages of preferred choice exhibited by WT, *MAPT* KI, *MAPT*^Int10+3^ KI, and *MAPT*^S305N; Int10+3^ KI mice. **h**, Percentages of movement pattern (Re-entry, Short side, Long side, Diagonal) displayed by WT, *MAPT* KI, *MAPT*^Int10+3^ KI, and *MAPT*^S305N; Int10+3^ KI mice. All experiments were performed with 14-16-month-old female mice (WT: n = 14, *MAPT* KI: n = 11, *MAPT*^Int10+3^ KI: n = 15, *MAPT*^S305N; Int10+3^ KI: n = 11). Data represent the mean ± SEM (two-way ANOVA with Tukey’s multiple comparison test). Source data

**Extended Data Fig. 10:**
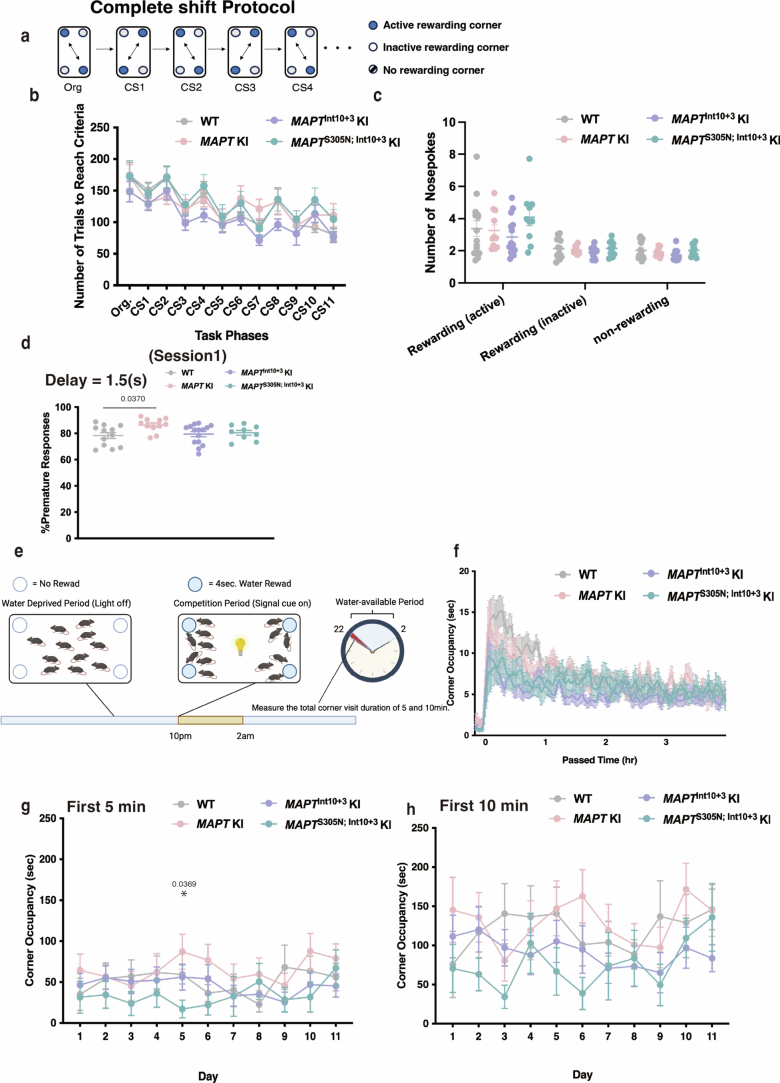
Results from SP-FLEX (behavioral sequence learning and behavioral flexibility task), behavioral inhibition and competitive dominance tests. **a-c**, Number of trials to reach criteria and nosepokes of 14-16-month old WT, *MAPT* KI, *MAPT*^Int10+3^ KI, and *MAPT*^S305N; Int10+3^ KI mice in the CS (complete shift)-only session of Self-paced Learning and Behavioral Flexibility Test (SP-FLEX) (WT: n = 14, *MAPT* KI: n = 11, *MAPT*^Int10+3^ KI: n = 15, *MAPT*^S305N; Int10+3^ KI: n = 10). **d**, Percentage of premature responses displayed by 14-16-month old WT, *MAPT* KI, *MAPT*^Int10+3^ KI, and *MAPT*^S305N; Int10+3^ KI mice in Session 1 of the behavioral inhibition test (WT: n = 12, *MAPT* KI: n = 11, *MAPT*^Int10+3^ KI: n = 15, *MAPT*^S305N; Int10+3^ KI: n = 9). **e,f**, Average corner occupancy time displayed by WT, *MAPT* KI, *MAPT*^Int10+3^ KI, and *MAPT*^S305N; Int10+3^ KI mice at 0-4 h over 11 days. (WT: n = 10, *MAPT* KI: n = 9, *MAPT*^Int10+3^ KI: n = 14, *MAPT*^S305N; Int10+3^ KI: n = 9) (created with BioRender.com). **g**, Average corner occupancy time displayed by WT, *MAPT* KI, *MAPT*^Int10+3^ KI, and *MAPT*^S305N; Int10+3^ KI mice in the first 5 min over 11 days. **h**, Average corner occupancy time displayed by WT, *MAPT* KI, *MAPT*^Int10+3^ KI, and *MAPT*^S305N; Int10+3^ KI mice in the first 10 min over 11 days. In (**d**), data represent the mean ± SEM (one-way ANOVA with Tukey’s multiple comparison test). In (**g**), data represent the mean ± SEM. (two-way ANOVA with Tukey’s multiple comparison test). Source data

To further assess behavioral phenotypes thought to reflect clinical symptoms in patients with FTD with tauopathy^44^, we conducted a series of behavioral tests using IntelliCage (TSE Systems/NewBehavior)^45–47^—an automated home-cage monitoring system for group-housed mice. Specifically, we focused on parameters assessing behavioral disinhibition, apathy, behavioral inflexibility/perseverative behavior and competitive dominance to assess whether the mice showed behavioral symptoms seen in FTD^48^. In the analysis of the 2-week basal activity in the IntelliCage, *MAPT*^Int10+3^ KI mice visited corners more frequently than other genotypes. No differences were observed for other parameters, including drinking activity (that is, the number of nosepokes and lickings), preference and movement patterns among corners, and nosepoke holes (Extended Data Fig. 9), suggesting that the genotypes show no severe behavioral problems that would substantially complicate the interpretation of subsequent IntelliCage experiments. In the self-paced behavioral sequence learning and behavioral flexibility task (SP-FLEX task), mice were required to learn a sequence of moving back and forth between two distant rewarding corners. To shift their behavior, the positions of the rewarding corners were changed. The number of trials to reach criterion in each task phase, a learning performance index, did not differ among all genotypes in either Session 1 or 2 (Fig. 8a,b and Extended Data Figs. 10a,b). However, in Session 2, a significant increase in the number of perseverative nosepokes in rewarded visits was observed in *MAPT*^S305N;Int10+3^ KI mice compared with WT, *MAPT* KI and *MAPT*^Int10+3^ KI mice, with a similar tendency in Session 1 (Fig. 8c and Extended Data Fig. 10c). The behavioral inhibition task was conducted to assess the ability of mice to control impulsiveness. In this task, mice were required to withhold nosepoking for a certain amount of time (‘delay time’) to obtain a water reward in each trial. A nosepoke observed during the delay time was counted as a premature response—an index of impulsivity. It has been shown that, when a longer delay time is presented, the premature response rate is higher^49^. Three sessions (Sessions 1–3) were conducted, with the average delay time increasing as the sessions progressed (Fig. 8d). The percentage of premature responses of *MAPT*^Int10+3^ and *MAPT*^S305N;Int10+3^ KI mice was significantly increased compared with WT mice, specifically in Sessions 2 and 3, where the average delay time was longer (Fig. 8e,f). This effect was also observed in *MAPT* KI in Session 1 (Extended Data Fig. 10d). These results suggest that *MAPT*, *MAPT*^Int10+3^ and *MAPT*^S305N;Int10+3^ KI mice show behavioral disinhibition, described as ‘impulsive, rash or careless actions^47^.’ The taste preference and effort-based choice test, using saccharin dissolved or plain water as rewards of different value, was conducted to assess anhedonia and/or apathy-like behavioral signs. All genotypes showed a general preference for the sweet taste of saccharin (Fig. 8g,h). However, *MAPT*^S305N;Int10+3^ KI mice showed signs of elevated effort aversion, indicated by the significantly decreased rate of saccharin water choice in the subsequent test, where incremental effort (repetitive nosepoking) was required as a cost of receiving brief access to saccharin water (Fig. 8i). These results indicate behavioral signs of apathy in *MAPT*^S305N;Int10+3^ KI mice. Finally, the competitive dominance test was conducted to assess social behavior in competing for a limited water reward supply (that is, four corners) after a restriction period. Results showed no significant difference in competitive dominance among genotypes through the sessions (days), while *MAPT*^S305N;Int10+3^ KI mice showed a relatively reduced level of the dominance index, a corner visit duration in the competitive timeperiod (the first 5 and 10 min) of the test (Extended Data Fig. 10e–h).

## Discussion

Here we describe the generation of mouse models of human FTD where isoforms of human tau are expressed at physiological levels, closely recapitulating the spatiotemporal expression of tau observed in the brains of patients with 4R tauopathies. The insertion of FTD splice-shifting mutations at S305N and Int10+3 allows us to study the earliest changes associated with an increase in 4R-tau, focusing on hyperphosphorylation and tau aggregation.

Although these two mutations are rare in the human population (and they do not co-occur), they are both thought to lead to a relatively prevalent primary tauopathy through the same pathway—an increase in the level of 4R-tau^50^. We have shown that expression of the two mutations in *MAPT* KI mice resulted in the generation of predominantly 4R-tau, with total tau levels remaining unchanged. The *MAPT*^Int10+3^ and *MAPT*^S305N;Int10+3^ KI lines displayed similar phenotypes, but they were exacerbated in the *MAPT*^S305N;Int10+3^ KI line corresponding with an increase in 4R:3R-tau. Both lines showed an elevated level of phosphorylated tau compared with the nonmutant *MAPT* KI control. Tau phosphorylated at several epitopes was distributed diffusely and, in some neurons, it accumulated in the soma, with the strongest signal being seen in the hippocampus and entorhinal cortex. Synapse loss and early signs of neurodegeneration (amino-cupric silver staining) were seen in the *MAPT*^S305N;Int10+3^ KI line. Behavioral tests indicated that the *MAPT*^S305N;Int10+3^ KI line developed behavioral abnormalities, which were characterized by apathy, behavioral disinhibition and memory loss. Of note, all of these phenotypes occurred in the absence of detectable higher-order seed-competent tau structures.

The accumulation of hyperphosphorylated tau is consistently associated with pathological lesions in human AD and FTD post mortem material, and it has been associated with pathology and toxicity in numerous animal studies^51^. The relationship between splice isoform accumulation and pathological phosphorylation of tau is complicated as hyperphosphorylated tau is a feature of not only 4R tauopathies but also diseases in which the 3R:4R ratio is maintained (AD) or shifted to 3R (Pick’s disease). Our data indicate that increased 4R-tau is associated with the early accumulation of hyperphosphorylated tau in the *MAPT* KI mutant mice. Phosphorylation/hyperphosphorylation is thought to destabilize the association of tau with microtubules, leading to its relocation from axons to the soma and dendritic compartments^9,52,53^. Given that our mice have physiological levels of tau and they do not have overt neurofibrillary pathology, our data support a causal role for hyperphosphorylated tau in pathogenic mechanisms associated with neurotoxicity. However, the exact mechanism(s) linking 4R-tau and hyperphosphorylation, and the impact hyperphosphorylated tau might have on microtubule stability, tau distribution, synaptic integrity and cognitive performance in mice needs to be investigated further. A range of *MAPT* KI mutant mice modelling these aspects of human tauopathies will be invaluable to address pathomechanistic questions.

The data provided in this study shows that the mutant *MAPT* KI mice recapitulate some of the spatial and morphological aspects of the human neuropathology associated with FTLD-*MAPT*-S305N and Int10+3 mutations. Macroscopically, two reports of S305N mutation carriers described pathological inclusions in the hippocampus, amygdala and thalamus, which is in line with our results^28^. S305N carriers show predominantly somatic, perinuclear accumulation of phosphorylated tau mainly in the outer granular layer of the shallow cortical layer, rather than the flame-shaped tangles commonly seen in human AD. Interestingly, most of the 4R-tau inclusions described in the FTLD-S305N mutation carriers resemble those found in the 3R tauopathy Pick’s disease. A similar spatial pattern has also been described for Int10+3 mutation carriers^54,55^. One study described inclusions as ‘pretangles,’ that is, diffuse granular distribution of hyperphosphorylated tau in the soma and dendrites^56^, which is similar to the inclusions described in this study.

Previous work has linked soluble phosphorylated tau to synaptic loss in AD, reinforcing the idea that soluble tau species, separate to classic tangles, can be related directly to neuronal damage and dementia^57^. The phosphorylation of particular epitopes, especially in the proline-rich domain, has been linked to synaptic dysfunction through the mislocalization of phospho-tau to dendritic spines^53^. It is not known exactly how the accumulation of hyperphosphorylated tau is linked to synaptotoxicity but its role in stabilizing microtubules has been implicated, as tau is thought to detach from microtubules as a result of hyperphosphorylation of epitopes in the proline-rich domain and C-terminus of the tau protein^9^. The destabilization of microtubules has been linked to altered pre- and postsynaptic structure and function^53^. Several of the same epitopes implicated in microtubule stabilization are hyperphosphorylated in the mutant *MAPT* KI mice. Published studies have indicated that tau is relocated from the axon to the dendritic spine following hyperphosphorylation. This has been proposed to lead to downregulated AMPA (α-amino-3-hydroxy-5-methyl-4-isoxazolepropionic acid receptor) and NMDA (N-methyl-d-aspartate) receptor expression on the postsynaptic membrane, which was shown to dramatically impair synaptic function^53^. It is therefore feasible that hyperphosphorylation of tau in our mice could be related directly to synaptic dysfunction and loss. Synaptic loss was demonstrated in the *MAPT*^S305N;Int10+3^ KI mice; however, this was significant only when assessed by quantitative immunohistochemistry of adjacent pre- and postsynaptic compartments in situ, and not by immunoblotting of synaptic proteins extracted from intact blocks of tissue. We consider the former technique to be more rigorous and sensitive as it identifies only viable synapses, and it preserves spatial information.

Overall, data from independent, orthogonal experiments using complementary methods did not reveal the presence of higher-order tau oligomers, aggregates or misfolded tau conformers in either mutant *MAPT* KI line at the ages studied. Following sedimentation through the sucrose gradient and sarkosyl extraction, the presence of tau species in the 10% fraction could reflect the formation of low-number oligomers (<hexamer)^36^ or cosedimentation of tau with an interactor. The presence of sarkosyl-insoluble tau does not necessarily represent aggregated tau as insolubility could be driven by other mechanisms, such as condensation^58^, rather than self-aggregation. Immunohistochemistry using the conformation-specific antibody MC1 did not reveal accumulated misfolded tau conformers or either of the mutant *MAPT* KI lines induced seeding in the biosensor assay. Post mortem tissue from a human S305N mutation carrier did show immunoreactivity for the MC1 antibody in the soma of cells resembling neurons but the signal seemed to be reduced compared with other pathological tau antibodies. In support of the MC1 data, brain extract from the human S305N mutation carrier seeded the S305N biosensor, confirming the presence of conformationally abnormal, seed-competent tau in the human tissue. As the human tissue was from a donor with late-stage disease, it is possible that our *MAPT* KI mutant mice would develop seed-competent tau with further aging; however, our results from RT-QuIC and MC1 staining at more advanced age suggest that this will not be the case, indicating a limitation of the mice which should be considered to best model the earliest stages of tauopathy. It is interesting to note that the S305N *MAPT* human tissue induced seeding in the S305N but not in the P301S cell line developed by the Diamond laboratory^37^. This suggests that a conformation barrier exists between S305N and P301S tau and it reinforces the notion that it is important to use the correct homotypic biosensor cells to measure the seeding activity of tau appropriately. The potential for a seeding barrier was supported by the observation of reduced seeding efficiency in the S305N biosensor compared with the P301S biosensor line by tau extract from the PS19 mouse control (P301S *MAPT* mutation), although technical issues, such as lower levels of expression of the S305N biosensor construct compared with the P301S construct, could also account for this difference. Also of note was the observation that tissue from human AD and Int10+3 mutation carrier FTD cases did not seed in the S305N biosensor, which again supports the idea of a conformational seeding barrier. Both the S305N and the Int10+3 mutations affect 4R splicing, and tau isolated from late-stage material from patients is in the form of flat ribbons^3,28,59^. However, there is no evidence that the conformation of human tau in patients with S305N and Int10+3 is the same, as the structure of S305N tau has not been resolved by sensitive techniques such as cryo-EM, due to the lack of suitable archived brain tissue and the scarcity of mutation carriers. Finally, RT-QuIC data shows that the random templating detected in the *MAPT* KI and *MAPT*^S305N;Int10+3^ KI indicates the lack of higher-order structures present in the mouse brain homogenates, even at a later age of 24 months. Although all reactions were considered to be random, the possible differences in reaction time and ThT amplitude between the *MAPT* KI and *MAPT*^S305N;Int10+3^ KI could be explained by the differences in isoforms^38,39,60^, or tau structures induced by the double mutation and/or hyperphosphorylation; however, this needs further assessment. Despite some of the limitations of our studies, it is still possible to conclude that large oligomers or fibrillar aggregates were not necessary to trigger synaptic loss and neurodegeneration in our mouse models.

Episodic memory problems have been described widely in patients with FTD and they are part of the current diagnostic criteria^61^. In the *MAPT*^S305N;Int10+3^ mouse line, behavioral tests focusing on memory showed a reduction in spatial memory. To identify more subtle behavioral abnormalities, we used the IntelliCage system, which revealed perseverative behavior in the *MAPT*^S305N;Int10+3^ K mice. Repetitive behavior, including simple repetitive movements, complex behaviors or stereotypy in speech, and disinhibition, specifically socially inappropriate behavior, loss of manners or decorum and impulsive, rash or careless actions, are at the core of the diagnostic criteria for the behavioral variant of FTD (bvFTD), which affects around half of the cases of FTD^62^. There was some evidence that humanization of the *Mapt* gene could have an effect on some of the behaviors tested as a significant impulsive behavior was detected in *MAPT* KI mice (as well as the mutants) compared with WT, and the saccharin choice in *MAPT* KI mice on the effort-based choice test seemed to be slightly lower compared with WT mice. Humanization of the *Mapt* gene could therefore be associated with impulsivity and aspects of apathy—a phenotype consistent with the recent clinical finding in patients with bvFTD, where effort avoidance is more pronounced, while reward appetence is less affected^63^. The impact of humanization alone may have masked any additional effects of mutations in impulsive and motivation measures. Further assessments will be required to understand how humanization of tau could affect these particular behavioral measures. In the classical behavior tests, although *MAPT* KI mice displayed hyperactivity in the open field compared with WT mice, which is in agreement with previous studies^64^, locomotive parameters in other classical behavior assays were comparable across the genotypes. This evidence supports the idea that locomotion activity was not a confounding factor in the interpretation of cognitive performance in *MAPT*^S305N;int10+3^ mice. Although the difference in behavior between genotypes is subtle at the age tested and further analysis is warranted, especially at advanced ages, behavioral differences do support our data showing significant synapse loss and neuritic degeneration.

In conclusion, here we provide evidence that synaptic loss and behavioral abnormalities occur independently of seed-competent higher-order structures, which are not needed to initiate toxic pathological mechanisms. The value of this study lies not only in the identification of the earliest events in disease pathogenesis, but in the provision of physiologically relevant mouse models of tauopathies that will aid in our understanding of how to prevent neurodegeneration and in the development of therapeutic strategies.

## Methods

### Animals

All animal experiments were conducted in accordance with guidelines of the RIKEN Center for Brain Science (W2021-2-020(3)) and UK Animal Act, 1968 (PP7490525), and following local ethical advice. C57BL/6J and ICR (Jcl) mice were used as zygote donors and foster mothers. C57BL/6J mice were also used for backcrossing with *MAPT* mutants. *MAPT* KI mice were generated as previously described^19,65^.

### Human tissues

Human brain samples were kindly provided from NHO Iou National Hospital in Japan, Queen’s Square Brain Bank (QSBB) in the UK and The Mayo Clinic in the USA. Brain samples were donated in compliance with the 1998 Data Protection Act, and they are summarized in Supplementary Table 5. All patient tissues were donated with full informed consent. Ethical approval for the study was acquired from NHS and UCL ethics committee (QSBB UCLMTA06-23) and in accordance with the Human Tissue Authority’s code of practice and standards, with an approved material transfer agreement, where relevant.

### Antibodies

Antibodies used in this research are listed in Supplementary Table 3. The specificity of the tau antibodies was confirmed using the *Mapt* KO mouse.

### Preparation for BEs and sgRNAs

In this study, Base Editor (BE), which is a fusion protein composed of *Streptococcus pyrogenes* Cas9 (SpCas9) and rat APOBEC1 (apolipoprotein B mRNA-editing enzyme, catalytic polypepide-like1)^29^, and VQR (D1135V, R1335Q and T1337R)-BE of which the protospacer adjacent motif (PAM) is changed from the NGG of the original SpCas9 (ref. ^66^) to NGA, were used for the introduction of FTLD-associated mutations into the *MAPT* gene of *MAPT* KI mice. For the synthesis of BE and VQR-BE mRNA in vitro, plasmid vectors pCMV-BE3 (Addgene, plasmid cat. no. 73021) and pBK-VQR-BE3 (Addgene, plasmid cat. no. 85171) were obtained from Addgene. To prepare the mRNA templates for BE and VQR-BE, PCR was performed with Herculase II Fusion DNA Polymerase (Agilent Technologies, cat. no. 600675). BE and VQR-BE were then synthesized with the mMESSAGE mMACHINE T7 Ultra Transcription kit (Thermo Fisher Scientific, cat. no. AM1345). Templates for the in vitro transcription of sgRNAs were also synthesized by PCR with Herculase II Fusion DNA Polymerase as described previously^5^. The MEGAshortscript T7 (Thermo Fisher Scientific, cat. no. AM1354) and MEGAclear (Thermo Fisher Scientific, cat. no. AM1908) kits were used for in vitro transcription of sgRNAs, while the CRISPR Design tool was used for creating sgRNAs^67^. Oligonucleotide sequences used for the in vitro transcription of BE, VQR-BE and sgRNAs are listed in Supplementary Table 4.

### Microinjection of mouse zygotes

BE and VQR-BE mRNA (60 ng µl^−1^) and sgRNAs (200 ng µl^−1^) were injected into the cytoplasm of *MAPT* KI zygotes. After incubation at 37 °C for 24 h, embryos developed to the two-cell-stage were transplanted into host ICR mice.

### Off-target analysis

Off-target sites that accepted up to three mismatches were determined by COSMID (https://crispr.bme.gatech.edu/)^32^. Target sites were amplified from tail genomic DNA by PCR using the Ex Taq-Polymerase kit (Takara, cat. no. RR001A), with primers listed in Supplementary Table 4. Target sequencing was performed using a DNA sequencer (ABI 3730xl).

### Whole-genome resequencing

#### Library construction

DNA samples extracted from mouse tail were prepared according to the Illumina TruSeq DNA sample preparation guide to obtain a final library of 300–400 bp average insert size. Genomic DNA (100 ng) was fragmented by the Covaris system with specific tubes, which generates double-strand DNA fragments with 3′ or 5′ overhangs. Fragmented DNAs were then converted into blunt ends using an End Repair Mix. The 3′ to 5′ exonuclease removes the 3′ overhangs, and the polymerase fills in the 5′ overhangs. Following the end repair, the appropriate library size was selected using different ratios of sample purification beads. Adenylation of 3′ ends was performed to prevent ligation during the adapter ligation reaction. A corresponding single ‘T’ nucleotide on the 3′ end of the adapter provides a complementary overhang for ligating the adapter to the fragment. Several indexing adapters were ligated to the ends of the DNA fragments to prepare them for hybridizing onto a flow cell. PCR was used to amplify the enriched DNA library for sequencing. The PCR was performed with a PCR primer solution that annealed to the ends of each adapter.

#### Clustering and sequencing

For cluster generation, the library was loaded into a flow cell where fragments were captured on a lawn of surface-bound oligonucleotides complementary to the library adapters. Each fragment was then amplified into distinct clonal clusters through bridge amplification. When cluster generation was complete, the templates were ready for sequencing. Illumina SBS technology utilizes a proprietary reversible terminator-based method that detects single bases as they are incorporated into DNA template strands. As all four reversible, terminator-bound dNTPs were present during each sequencing cycle, natural competition minimized incorporation bias and greatly reduced raw error rates compared with other technologies. The result was a highly accurate, base-by-base sequencing that virtually eliminated sequence-context-specific errors, even within repetitive sequence regions and homopolymers.

#### Generation of raw data

The Illumina platform generates raw images and base calling through an integrated primary analysis software called RTA (real-time analysis). The BCL (base calls) binary files were converted into FASTQ files using the Illumina package bcl2fastq2-v.2.20.0.

#### Sequencing analysis

Resulting leads were aligned using Isaac Genome Alignment software (v.01.15.02.08), which is designed to align next-generation sequencing data with low-error rates^68^, and Isaac Variant Caller (v.2.0.13), which is used for identifying single-nucleotide variants and small insertions and deletions in the diploid genome case. The annotation tool SnpEff was used to categorize the effect of variants on genes^69^.

#### RNA extraction and semiquantitative RT-PCR

Total RNA was extracted from mouse brain cortices using RNAiso Plus (Takara cat. no. 9109) according to the manufacturer’s instructions. Reverse transcription was performed using ReverTra Ace (TOYOBO FSQ-301). Semiquantitative RT-PCR was conducted using the QuantStudio system (Thermo Fisher Scientific). Primer pairs are listed in Supplementary Table 4. The specificity of tau primers was confirmed using the *Mapt* KO mouse.

#### Western blot analysis after alkaline phosphatase treatment

Mouse brains were homogenized in lysis buffer (50 mM Tris pH 7.6, 0.15 M NaCl, cOmplete protease inhibitor cocktail (Roche Diagnostics #11697498001)) using a Multi-bead shocker MB (Yasui-Kikai). Samples were rotated at 4 °C for 1 h and centrifuged at 19,800*g* for 30 min. Supernatants were collected as lysates and then subjected to sodium dodecyl sulfate–polyacrylamide gel electrophoresis (SDS–PAGE), followed by transfer to a PVDF membrane. For detection of the tau isoform pattern, brain lysates were dephosphorylated with lambda-phosphatase (Santa Cruz Biotechnology, cat. no. sc-200312A) according to the manufacturer’s protocols. Membranes were then treated with ECL prime blocking buffer (GE Healthcare, cat. no. RPN418) for 1 h and incubated with antibody at 4 °C. Dilution ratios of antibodies are listed in Supplementary Table 3. Immunoreactive bands were visualized with ECL Select detection reagent (GE Healthcare, cat. no. RPN2235) and a LAS-3000 Mini Lumino image analyzer (Fujifilm). Image J (v.1.53a) and Fiji software v.1.0 (NIH) were used for data analysis.

#### LC–MS/MS analysis targeting tau peptides

According to the procedure detailed in Lantero-Rodriguez et al.^70^, Brains were homogenized in Tris-buffered saline (containing protease inhibitor cocktail) and centrifuged at 4 °C for 1 h at 31,000*g*. Protein quantification was performed on the supernatant. To immunoprecipitate tau protein, 4 μg of the monoclonal antibody HT7 (Thermo Fisher Scientific) was conjugated with 50 μl M-280 Dynabeads (Invitrogen) according to the manufacturer’s practice. HT7-coated beads were used to immunoprecipitate 10 μg of total protein from brain extracts and all samples were brought to a final volume of 1 ml with PBS with 0.05% Triton X-100. To each sample, 10 µl of protein standards mix (^15^N-labelled full-length tau and ^13^C- and ^15^N-labeled phosphorylated peptides at lysine or arginine). To have a full coverage of all peptides reflecting tau isoforms, a mix of 0N3R, 1N4R and 2N4R was selected (133 fmol of each isoform). Samples were subsequently incubated overnight at 4 °C on a rolling station. Following bead washing, elution was achieved with 100 μl of 0.5% formic acid. Eluates were collected and dried in a vacuum centrifuge. Quantification of phospho-sites synthetic phosphorylated peptides stable isotope-labeled at Lys or Arg matching definite trypsin cleavage sites (Thermo Fisher Scientific) were added as internal standards. The labelled peptides were diluted, mixed and 100 fmol was added to each sample before trypsination, which was performed by adding 100 ng trypsin (Promega) in 50 mM ammonium bicarbonate. Samples were brought to a final volume of 50 μl with ammonium bicarbonate and shaken overnight at 100 RPM at 37 °C. Following trypsination quenching with 10% formic acid, samples were dried in a vacuum centrifuge. Before nanoflow LC–MS analysis, samples were reconstituted in 7 µl of 8% acetonitrile/8% formic acid. A Dionex 3000 LC-system (Thermo Fisher Scientific) was coupled to an electrospray ionization hybrid quadrupole-orbitrap mass spectrometer (Q Exactive, Thermo Fisher Scientific). For each sample, 6 µl was loaded onto a reverse-phase Acclaim PepMap C18 trap column (Thermo Fisher Scientific) for desalting and sample clean-up. Separation was performed with a reversed-phase Acclaim PepMap C18 analytical column (Thermo Fisher Scientific) by applying a 50 min linear gradient from 3% to 40% B at a flow rate of 300 nl min^−1^; buffer A was 0.1% formic acid and buffer B was 84% acetonitrile/0.1% formic acid. The mass spectrometer was operated in positive ion mode using data-dependent acquisition so that for each full mass scan, up to five MS/MS scans followed. Fragmentation was performed using higher-energy collisional dissociation (HCD). Acquisition settings for both full scans and MS/MS scans were: resolution 70,000, one microscan, target value 10^6^, injection time 250 ms; for MS/MS the normalized collision energy was set at 25. LC–MS data were searched against a custom-made tau database using Mascot Deamon v.2.6/Mascot Distiller v.2.6.3 (Matrix Science) for charge and isotope deconvolution before the search using the Mascot search engine. Quantitative analysis was performed using Skyline v.20.1.0.31 (MacCoss laboratory) on the LC-traces from the full mass scans. Every peak was inspected manually and adjusted when required. The ratio between the endogenous peak and the heavy-labeled standard was used to get a relative peptide quantification.

#### Tissue fixation and preparation for FFPE and free-floating staining

Brains were perfused in PBS and 4% paraformaldehyde (Nacalai Tesque, cat. no. 09154-85) and embedded in paraffin. For free-floating staining, following perfusion, brains were immersed in 30% sucrose/PBS for 48 h at 4 °C and embedded in optimal cutting temperature compound (Sakura Finetek, cat. no. 4583).

#### Immunohistochemistry and immunofluorescence

For immunostaining of several tau antibodies, 30 µm free-floating sections were washed in PBS, followed by endogenous peroxidase quenching in 0.3% H_2_O_2_ in methanol for 15 min at room temperature. Sections were blocked for 1 h 30 min in M.O.M. blocking solution (Vector laboratories) and incubated in primary antibody overnight at 4 °C (Supplementary Table 3). Sections were washed in PBS, followed by 15 min secondary antibody incubation and 10 min signal-amplification with avidin-biotin complex (Vector Laboratories) incubation. Positive signal was detected using 3,3′-diaminobenzidine activated with H_2_O_2_. For signal quantification, five 30 µm horizontal sections were selected from each mouse brain at the following bregmas (according to Paxinos and Franklin mouse brain atlas): −1.24 mm, −2.36 mm, −3.28 mm, −4.28 mm and −5.36 mm. For each bregma *N* = 6 mice were stained (*N* = 5 for nontransgenic WT mice). All sections were stained together, blinded to genotypes. A score from 0 to 2 was added by visually assessing all the anatomical regions on the atlas. Scoring was performed by two independent scientists. Data were imported on Prism for heatmap generation, and anatomical representative images were performed using BrainGlobe-Heatmap^71^. Labels were added manually.

For immunostaining of Homer1, synaptotagmin and vGLUT1, 30 μm free-floating tissue sections were pretreated in 1% Triton X-100 for 20 min. Sections were then blocked in 20% NGS, 1% BSA and 0.3% Triton, in PBS for 2 h followed by standard primary antibody incubation overnight at 4 °C. Sections are washed in 0.3% Triton X-100 in PBS for 30 min followed by secondary incubation for 4 h at room temperature and washed for 30 min before mounting.

For human FFPE staining, 8 µm sections were deparaffinized in xylene and rehydrated using graded alcohols. Following pressure cooker pretreatment in citrate buffer for 10 min and endogenous peroxidase quenching for 10 min, sections were blocked in 10% dried milk solution. Tissue sections were incubated with primary antibodies for 1 h at room temperature, followed by biotinylated secondary incubation for 30 min at room temperature and avidin-biotin complex for 30 min. Color was developed with 3,3′-diaminobenzidine/H_2_O_2_.

For paraffin-embedded mouse brain sections, antigen retrieval was performed by autoclaving sections at 121 °C for 5 min after deparaffinization. Sections were then treated with 0.3% H_2_O_2_ in methanol to inactivate endogenous peroxidases, which was followed by rinsing several times with TNT buffer (0.1 M Tris pH 7.5, 0.15 M NaCl, 0.05% Tween-20), blocking for 30 min (TSA Biotin System kit), and incubation overnight at 4 °C with primary antibody diluted in TNB buffer (0.1 M Tris pH 7.5, 0.15 M NaCl). Sections were rinsed several times and incubated for 1 h at room temperature with biotinylated secondary antibody (Vector Laboratories). Next, sections were incubated with horseradish peroxidase (HRP)-conjugated avidin for 30 min and tyramide-enhanced fluorescein isothiocyanate or rhodamine for 10 min. Finally, sections were stained with 4,6-diamidino-2-phenylindole (Cell Signaling Technology, cat. no. 4083S) diluted in TNB buffer before mounting with PermaFluor (Thermo Fisher Scientific, cat. no. TA-030-FM). Sections were scanned on a NanoZoomer Digital Pathology C9600 (Hamamatsu Photonics) and analyzed by the Definiens Tissue Studio (v.3.60).

#### Gallyas silver staining

The sections were deparaffinized and hydrated with xylene and ethanol and placed the sections in 5% periodic acid for 3–5 min. Then, the sections were rinsed with H_2_O and placed in silver iodide solution (300 ml H_2_O containing 12 g sodium hydroxide, 30 g potassium iodide, and 10.5 ml 1% silver nitrate) for 1 min. The sections were placed in 0.5% acetic acid for 10 min and rinsed with H_2_O. The sections were placed in the solution containing 200 ml of 5% anhydrous sodium carbonate, 100 ml of buffer (0.19 g ammonium nitrate, 0.2 g silver nitrate, 1 g tungstosilicic acid in H_2_O) and 100 ml of buffer (0.19 g ammonium nitrate, 0.2 g silver nitrate, 1 g tungstosilicic acid and 0.76 ml 37% formaldehyde in H_2_O) and stopped development in 0.5% acetic acid for 5 min, followed by dehydration with ethanol and Xylene.

#### Sucrose gradient separation

The sucrose gradient separation of tau oligomers was adapted from Jackson and colleagues^36^. Briefly, mice at 6 and 18 months were euthanized by cervical dislocation and decapitation. Whole brains were homogenized in 1.5 ml ice-cold PBS with HALT Protease and Phosphatase inhibitor cocktail (Thermo Fisher Scientific, cat. no. 78441) using a dounce homogenizer. Homogenates were spun down at 13,000*g* for 10 min at 4 °C. The supernatants were set aside, and the pellet was re-extracted a further three times, being careful not to exceed a total volume of 3 ml. Protein concentrations were determined using a bicinchoninic acid assay (Thermo Fisher Scientific cat. nos. 23228 and 23224); 2.5%, 5%, 10% and 20% sucrose were prepared in water and layered onto 13.2 ml ultraclear centrifuge tubes (Beckman, cat. no. 344059) in decreasing density (4 ml of 20% sucrose and 1.5 ml of 10%, 5% and 2.5% each). Equal amounts of total protein in 3 ml from each mouse brain lysate were added on top of the gradient. The prepared gradients were then spun at 250,000*g* for 4 h at 4 °C in a SW41Ti rotor (Beckman), with no breaking during deceleration. Six fractions were collected and stored at −80 °C until analysis. The PBS fraction remaining at the top of the sucrose gradient following centrifugation was called ‘Top.’ The tube pellet that formed was sonicated into PBS for 3 min at 50% amplitude (30 s on, 15 s off) at 4 °C and saved at −80 °C. For analysis of Sarkosyl insolubility, 1 ml of each fraction was incubated with 1% final volume of Sarkosyl for 1 h at room temperature with gentle rotation. Samples were then centrifuged for 1 h at 100,000*g* in a MLA-55 rotor. The pellet, containing sarkosyl-insoluble tau, was sonicated into PBS for 1 min at 60% amplitude (15 s on, 5 s off) at 4 °C.

#### Western blot analysis of sucrose gradient separation

Total tau and sarkosyl-insoluble pellets from each fraction were prepared in 4× Laemmli sample buffer (Bio-Rad, cat. no. 1610747) containing 355 mM 2-mercaptoethanol, and boiled at 95 °C for 5 min. Samples were resolved using 7.5% Criterion TGX Stain-Free gels (Bio-Rad). Proteins were transferred to low-fluorescence PVDF membranes (Bio-Rad) and visualized using ultraviolet light in a Chemi-Doc MP imager (Bio-Rad). Membranes were blocked with 5% milk powder in Tris-buffered saline (20 mM Tris-HCl pH 7.4, 150 mM NaCl) containing 0.02% Tween-20 (TBST) for 1 h at room temperature. Primary antibodies were diluted in Superblock TBS blocking buffer (Thermo Fisher Scientific) and incubated with the membrane overnight at 4 °C. Membranes were then washed twice in TBST and twice in 5% milk powder for 10 min each. Secondary HRP-conjugated secondary antibodies were diluted in 5% milk powder and incubated with the membrane for 1 h at room temperature. Membranes were then washed four times in TBST for 10 min each. Subsequently, proteins were visualized using Bio-Rad Clarity Max Chemiluminescent substrate (cat. no. 1705062) in a Chemi-Doc MP imager. The total protein signal, visualized using ultraviolet light, was used to normalize labeled protein signal using the Image Lab software package v.6.1, build 7 (Bio-Rad). Dilution ratios of antibodies are listed in Supplementary Table 3. This experiment was repeated at least three times with consistent observations.

#### Processing of human FFPE tissue for seeding analysis

From each case, five sections of 8 μm thickness were cut on a microtome (HistoCore Biocut, Leica). Antigen retrieval was performed by autoclaving for 5 min at 121 °C. Gray matter from each of the five sections was scraped off the glass slide, pooled and placed into 10% w/v EDTA buffer (10 mM Tris, pH 8.0, 1 mM EDTA). Samples were heated for 25 min at 95 °C and allowed to cool down at 4 °C for 15 min. The samples were then sonicated with a water bath sonicator (Qsonica, cat. no. Q800R3) at 4 °C at 50 amplitude for 60 min and stored at −80 °C until further use.

#### Processing of mouse fresh frozen tissue for seeding analysis

The posterior cortex of one hemibrain was homogenized using a manual homogenizer (Kimble Pellet Pestle Cordless motor) in 10% w/v ice-cold TBS (50 mM Tris-HCl, pH 7.5, 150 mM NaCl) containing cOmplete protease inhibitors (Roche). Samples were sonicated with a water bath sonicator (Qsonica Q800R3) at 4 °C for 5 min at 65 amplitude (30 s on, 60 s off). Lysates were centrifuged at 21,000*g* for 15 min to remove cellular debris. Protein concentrations were determined using a bicinchoninic acid assay (Thermo Fisher Scientific, cat. no. 23228 and 23224). Supernatants were aliquoted and stored at −80 °C until further use.

#### Transduction of biosensor cell lines, flow cytometry and seeding analysis

HEK Tau RD P301S FRET biosensors (ATCC CRL-3275) or HEK Tau RD S305N-YFP biosensors were cultured in DMEM (Thermo Fisher, cat. no. 41966-029) with 10% FBS and 1% Pen-Strep. Cells were plated at a density of 38,000 cells per well in a 96-well plate in a volume of 130 µl medium per well. Cells for imaging were plated in a PhenoPlate 96-well microplate (Perkin Elmer) coated with poly-l-ornithine (Sigma-Aldrich, cat. no. P4957). At 18 h later, cells were transduced with proteopathic seeds; 1.25 µl lipofectamine 2000 (Invitrogen) was mixed with 8.75 µl Opti-MEM (Gibco, Life Technologies) and incubated at room temperature for 5 min before mixing with 10 µg of total protein for mouse samples or 4 µl for human samples. Negative controls received Lipofectamine in Opti-MEM. After incubating mixtures at 37 °C for 30 min, a final volume of 20 µl was added to each well, and cells were incubated for 48 h. For each sample, three technical replicates were included.

#### Flow cytometry

Tau RD P301S FRET biosensor cells were collected with 0.25% trypsin and fixed in 4% PFA for 10 min, then resuspended in flow cytometry buffer (1× PBS and 1 mM EDTA). An LSR Fortessa Flow Cytometer (BD Biosciences) was used to perform FRET flow cytometry on the Tau RD P301S FRET biosensors. FRET was quantified as described previously^72^. Briefly, single cells that were double-positive for YFP and CFP were identified and FRET-positive cells within this population were quantified. The gating strategy is described in Extended Data Fig. 6b. The percentage of FRET (the number of FRET-positive cells per total cell count) and the integrated FRET density (the product of percent positivity and median fluorescence intensity) were used as output measures. Data analysis was performed using FCS express v.7 (De Novo Software) and GraphPad Prism v.9.4.1 for Mac OS X.

#### Imaging and spot count analysis

Tau RD S305N-YFP biosensors were fixed in 4% PFA for 10 min and washed three times in DPBS. Cells were then stained with Hoechst (Invitrogen, cat. no. 33342) at 1:1,000 for 10 min at room temperature, washed three times in DPBS and left in PBS at 4 °C until images were acquired with the high-content confocal microscope Opera Phenix Plus (Perkin Elmer). Excitation wavelengths and emission filters used were as follow: YFP 488 nm, 650–760 nm, Hoechst: 375 nm, 435–480 nm. Images were obtained using a ×20 water immersive objective, with 12 fields of view per well, and a *Z*-stack of seven slices (1.5 μm). The images were analysed using Perkin Elmer software Columbus v.2.9.1. Briefly, images were loaded as maximum projection and basic flatfield correction was applied. Cells were segmented by finding nuclei using Hoeschst 33342 channel and cytoplasm was identified next by using the watershed of the Hoeschst channel. The YFP-positive tau inclusions were identified using the ‘finding spots’ building block (method D) on the YFP channel. The analysis result was exported and the number of spots per nuclei was calculated. Data were subsequently plotted in GraphPad Prism.

#### 4R-tau RT-QuIC

RT-QuIC was performed as described previously^38,39^. Briefly, K11CFh recombinant tau protein, a tau fragment comprising R1–R4 and extending to residue 400 with cysteine to serine mutations, was purified, and utilized for amplification of 10% w/v PBS (with protease and phosphatase inhibitors) extracts from human PSP frontal cortex and 24-month-old posterior cortex of *MAPT* KI and MAPT^S305N;Int10+3^ KI mouse samples. K11CFh was thawed from −80 °C and filtered through 100 kDa filters to remove preformed aggregates. Reactions were seeded with 1 × 10^−5^ concentration of brain homogenates in the presence of 3 μM K11, 10 μM ThT in a buffer containing 250 mM trisodium citrate,10 mM HEPES, pH 7.4. Reactions were subjected to rounds of 60 s shaking (500 rpm, orbital) and 60 s rest, with periodic ThT readings every 15 min at 37°C in a 384-well Nunc microplate (nontreated polymer base, cat. no. 242764) in a BMG FluoStar lite with aluminum sealing cover to prevent evaporation.

#### Super-resolution imaging and synapse analysis

Synapse counts were performed as previously described^73^. Super-resolution synapse images were obtained using a Zeiss LSM980 microscope with Airyscan detector with a ×63, 1.4 numerical aperture oil immersion Plan-Apochromat objective. The airyscan detector was aligned before imaging each new slide. Five regions of interest (ROIs) of 1.15 μm were obtained in the hippocampus and entorhinal cortex for each brain section. ROIs were identified by selecting the same bregma (according to the Paxinos horizontal mouse brain atlas, that is, bregma −3.76 mm). An overview of the whole hippocampus including the entorhinal cortex was performed under the 4,6-diamidino-2-phenylindole channel. For each bregma, the ROI at ×63 was taken in the following regions, trying to maintain the same location: polymorph layer of the dentate gyrus, radiatum layer of the hippocampus around the CA3 molecular layer (CA3-Rad), lacunosum molecular layer of CA1 (CA1-LMol) and layer II in the dorsolateral entorhinal cortex (EC-LyII). Super-resolution synapse images were processed in Zen Black using three-dimensional Airyscan Processing. Data were analyzed independently by two blinded investigators. Imaris software was then used for pre- and postsynaptic puncta detection, using intensity centers to maximize the detection of immunoreactivity spots. Pre- and postsynaptic spots were colocalized using a MATLAB colocalization script (Colocalize Spots XTension), using a colocalization distance of 0.25 μm between spot centers. Dilution ratios of pre- and postsynaptic antibodies are listed in Supplementary Table 3.

#### Amino-cupric silver staining

This method followed an original method described previously^43^. Preimpregnation solution (100 mg AgNO_3_, 100 ml H_2_O, 53 mg dl-*a*-amino-*n*-butyric acid, 46 mg dl-alanine, 2 ml 0.5% copper nitrate (Cu(NO_3_)_2_), 0.2 ml 0.5% cadmium nitrate (Cd(NO_3_)_2_), 1.5 ml 0.5% lanthanum nitrate (La(NO_3_)_3_), 0.5 ml 0.5% Neutral Red, 1.0 ml pyridine, 1.0 ml triethanolamine, 2.0 ml isopropanol) was heated in the microwave to 48 °C. Sections were placed into the preheated solution and cooled at room temperature for 2–3 h. Following, sections were placed in a porcelain filter and washed for 1 min with H_2_O and twice with acetone (45 s–1 min for each wash) to remove excess pyridine and preimpregnation solution. Tissue was then transferred in impregnation solution (5 ml H_2_O, 412 mg AgNO_3_, 4 ml 100% ethanol, 50 µl acetone, 3 ml 0.4% lithium hydroxide, 0.65 ml ammonium hydroxide) for 45–50 min. Sections were transferred for 20 min to the reduction solution (800 ml H_2_O, 90 ml 100% ethanol, 11 ml 10% formalin, 6.5 ml 1% citric acid monohydrate), which was preheated at 30 °C. Subsequently, the tissue was washed with H_2_O and placed into 0.25% acetic acid for 1 min to stop the reduction reaction. Sections were then washed with H_2_O for 1 min twice and the sections kept at 4 °C for at least 1 h before the bleaching treatment. Sections were then incubated in Solution 1 (6% potassium ferricyanide (K_3_[Fe(CN)_6_] and 4% potassium chlorate (KClO_3_ in lactic acid) in a porcelain desiccator at room temperature until they became relatively transparent (approximately 60 s) to remove background silver deposits but not degenerating elements, then washed with H_2_O. Sections were then transferred to Solution 2 (0.06% potassium permanganate KMnO_4_ and 5% sulfuric acid in H_2_O) in a porcelain desiccator and left until the sections became yellow (around 15–20 s). Sections were then placed into H_2_O and washed twice for 5 min each before the stabilization step; in this step, sections were transferred to stabilization solution (2% sodium thiosulfate (Na_2_S_2_O_3_) in H_2_O) for 1 min where they lost their yellow color and became transparent. Sections were washed with H_2_O for 5 min and placed in Rapid Fixer Solution (KODAK concentrated Rapid solution A + B, diluted 1:6 in H_2_O) for 1 min. Finally, sections were placed in H_2_O and mounted with DPX mounting medium (Merck, cat. no. 100579).

#### Behavior tests

Male mice (15–16 months of age) were used for classic behavior tests, while female mice (14–16 months of age) were used in the IntelliCage system. Male mice were not compatible with group-housing required for IntelliCage, and were therefore used for the remaining classic behavior experiments. Single-sex studies are a potential limitation of the study. The results of all behavior tests are summarized in the Extended Data Fig. 8g.

#### Y-maze

The Y-maze apparatus (O’Hara) was composed of gray plastic and consisted of three compartments (3-cm (width) bottom and 10-cm (width) top, 40-cm (length) and 12-cm (height) radiating out from the center platform (3 × 3 × 3 cm triangle). Each mouse was placed on one of the arms and allowed to explore freely for 8 min. A correct spontaneous alteration was counted when the mouse entered all three arms in consecutive entries with overlapping sets of three. This was used as a measure of short-term working memory performance^74^.

#### Open-field test

Each mouse was placed in the middle of an open-field maze (600 × 600 mm) (O’Hara) and allowed to explore the area for 10 min. The amount of time that mice spent in the central region was measured as an indicator of anxiety, whereas the total distance was measured as locomotion activity as previously described^75^. One subject from the WT mouse group was removed from the dataset in the analysis for total distance because the score was identified as an outlier.

#### New object location test

The day before Session 1 (first day), mice were placed in the middle of the box without any object and allowed to explore freely for 6 min as the habituation period. On the first day, two identical objects were placed inside the box, and mouse exploration for 6 min was recorded. After 24 h, in Session 2, mice were reintroduced into the box with the same two objects, one placed in an identical location and the other in a different location to that in Session 1. Characteristics of the exploratory behavior were again recorded for 6 min and analyzed as described in the next paragraph.

Discrimination index is calculated as time spent exploring the replaced object minus the time spent exploring the familiar object divided by total exploration time, which is not influenced by differences in exploration time^76^. If mice did not reach a 10-s minimum of exploration for both objects at 6 min, they were exclude from analysis, as this is not enough time exploring to learn/discriminate. We also used a cut-off of 55% to define the preference of the mouse towards new locations, as previously described^77^. Mice showing less than 55% preference towards new locations were considered to show no preference and therefore included in the failed population.

#### Barnes maze test

The Barnes maze test was performed using a circular open field with 12 holes around the perimeter (O’Hara); this was placed on a frame set to a final height of 90 cm above the floor. Each mouse was trained for 2 min (habituation), three times per day on two consecutive days to move via one of the holes from the brightly lit (800 lx) circular field to an escape box, composed of black Plexiglas (17 × 13 × 7 cm), without any visual cues. Acquisition training to analyze their learning ability also consisted of three trials daily at an intertrial interval of 30 min. Tracking of each mouse’s movements was recorded using video hardware (O’Hara). At the start of each trial, mice were placed in a start chamber located at the center of the circular open field. Subsequently, the start chamber was removed, and mice were allowed to explore the goal. The trial was terminated when mice accessed the escape box or when 5 min had passed. In the latter case, mice were compulsorily placed into the escape box and held there for 1 min. To avoid any olfactory bias, the apparatus was cleaned by wiping with 70% ethanol after each trial. Finally, the probe test to analyze their memory ability for 3 min was performed without the escape box 24 h after the final acquisition training to assess the memory parameter dependent on the spatial information based on environmental cues in the room. Four distal cues were placed around the circular open field during the whole test. Latency to reach the goal and the time spent around the goal were calculated at both the acquisition training and probe test.

#### Rotarod test

Motor performance was assessed with a Rotarod Treadmill (Muromachi KIkai Co., Inc., cat. no. MK-610A) according to the protocol as described with some modifications^78^. This task took place over two consecutive days. On the first day, the mice were placed on the rod for 2 min to be habituated to the environment. On the second day, mice were placed on the rod rotating 16 rpm, and the retention time was measured (maximal latency to fall is 300 s). The scores averaged with two trials were calculated as the mean latency to fall from the rod. To avoid any olfactory bias, the apparatus was cleaned by wiping with 70% ethanol after each trial. Two subjects from the WT mice group and one subject from the *MAPT*^S305N;Int10+3^ KI mice group were removed from the dataset in the analysis for latency to fall because the scores were identified as an outlier.

#### Principal component analysis

Behavior results were first scaled so that every variable had a mean value of 0 and an s.d. of 1, across experimental groups. PCA was performed, and variance axes (or PCs) were retained if efficient dimensionality reduction was displayed (individual variance > 1/number of input variables). Individual PC scores were projected in the two strongest variance vectors and averaged per experimental group to highlight broad behavior differences.

### IntelliCage system

#### Housing and testing environment

All behavior tests using IntelliCage (TSE Systems GmbH) were conducted at the animal facility of Phenovance LLC (Chiba, Japan), in accordance with that company’s Animal Care and Use Committee guidelines (experiment approval no. PRT202103_17RS). The animal facility was maintained at 20–26 °C, 40–70% relative humidity, 10–25 ventilation per hour and a 12 h light-dark cycle (08:00 on, 20:00 off). Two weeks before starting the IntelliCage experiments, mice were anesthetized by isoflurane inhalation with the aid of an SN-487-1T Scan instrument (Shinano Manufacturing) and implanted with a small, glass-covered radio-frequency identification (RFID) microchip via a disposable injector with needle (2.12 × 12 mm, FDX-B ISO11784/11785, FAREAD Technology). The mice were housed in conventional plastic cages (2000P type, 612 × 435 × 216 mm, Tecniplast) in groups of 12–13 animals until the experiment.

#### Apparatus (IntelliCage system)

IntelliCage is a fully automated testing apparatus for monitoring spontaneous and cognitive behaviors of group-housed RFID-tagged mice in a home cage^79^. The corners of the cage are equipped with four triangular operant chambers (hereafter referred as corner A, B, C and D) that permit the entry of a single mouse at a time. The RFID antenna at each corner entrance recognizes the microchip ID implanted in each mouse, while infrared sensors and licking sensors inside the corner chambers monitor mouse behavior. Each corner holds two water bottles, the nozzles of which can be accessed through two nosepoke holes with motorized doors; the infrared beam-break response detector on the nosepoke hole responds to the mouse nosepokes, which trigger door opening/closing. Experimenters can flexibly program the rules for opening/closing of the doors and the lighting pattern of three LEDs located above each nosepoke hole. Four IntelliCages were used for experiments and each group of mice was divided as evenly as possible (two to four mice per genotype per IntelliCage) for a total of 12 to 13 mice per IntelliCage. Each IntelliCage was usually cleaned once a week, at which time the general health of each mouse was also carefully checked (bodyweights were measured; condition of skin, hair, eyes, mouth, tail and basic walking movements in an open cage were assessed visually.

#### Programs used for IntelliCage experiments

For the following IntelliCage experiments, each task program was selected from the Phenovance Task Library, which contains a collection of task programs for IntelliCage created by Phenovance LLC using specialized software (Designer software, TSE Systems).

#### Exploratory behavior, basal activity and stereotypical behaviors

On the first day (Day 1) of the IntelliCage experiment, mice were transferred from the conventional 2000P cages to IntelliCages by tunnel handling^80^, 2 h into the light period (10:00). All doors in the corner chambers were kept open and mice were free to drink water from any corner. From 10:00 on Day 4, all the doors were closed to let mice acquire nosepoke behavior to access water. A single nosepoke opened the door for 3,000 ms to allow mice to drink water. The number of corner visits, nosepokes and lickings from Day 5 to Day 19 were measured as indices of basal activity and stereotypical behaviors (corner preferences, nosepoke hole preferences and movement patterns between corners).

#### Self-paced learning and behavioral flexibility test

This test is designed to assess mice’s learning ability of the behavioral sequence rule and behavioral flexibility to adapt to repetitive rule shifts (modified from an original protocol developed in a previous study^81^. Each mouse was assigned a pair of corners as rewarding corners and had to shuttle between them to obtain water as a reward; water could not be obtained from the other two corners (nonrewarding corners) until the rule switched. When a mouse nosepoked at one of the rewarding corners, the door opened for 2,000 ms to permit the water intake, with the following restrictions: (1) The door opened only once per visit and (2) a mouse was not permitted to obtain the reward by consecutively re-entering the same corner. Because of these restrictions, mice were forced to go back and forth between the assigned two rewarding corners to obtain the reward, one defined as the active corner and the other as the inactive corner. A success response was defined as obtaining a reward by a direct move from the previously active rewarding corner to the current active rewarding corner, while a failure response was defined as visiting one of the two nonrewarding corners and applying at least one nosepoke. The success and failure responses were detected automatically and input to Wald’s sequential probability ratio test (SPRT) statistics to calculate each mouse’s probability of success. The significance level for the acceptance of each criterion was 0.05. Corner visits without nosepokes were omitted from the SPRT calculation. Whenever the performance of a mouse reached the preset upper criterion (30%), the assignment of rewarding and nonrewarding corners for that mouse switched. Note that the switch occurred only for mice whose success rate reached the upper criterion of SPRT. The test consisted of two sessions called the ‘CS (complete shift)-only’ session and the ‘CS (complete shift)/PS (partial shift)-shuffled’ session, each of which lasted 24 h per day for 7 days. The detailed protocol for each session is described below (see also a previous study conducted using the same protocol)^82^.

Session 1 (CS-only session): in this session, the mice were required to learn a behavioral sequence in which they were rewarded for moving back and forth between two diagonally opposite corners (for example, corners A, C) out of four corners (corners A, B, C, D). When the performance reached the upper criterion of SPRT, the two corners that had been rewarding (corners A and C) and the two nonrewarding corners (corners B and D) were reversed. Note that the term CS means that all the previously rewarding corners and all the previously nonrewarding corners were reversed at the same time. Within a session, there were several test phases: the Original learning phase (Org) is the phase of initial learning of the behavioral sequence rule until the first reversal occurs; Org was followed by the CS1 phase, when the rewarding and nonrewarding corners assignments reversed for the first time; the phases proceed every time the reversal occurs (for example, CS2, CS3, and so on).

Session 2 (CS/PS-shuffled session): in this session, the same protocol as in the CS-only session was used until the end of the CS1 phase. When a mouse reached the upper criterion of SPRT in the CS1 phase, only one of the two rewarding corners was replaced by one of the two nonrewarding corners (henceforth referred to as PS). After that, the CS and PS phases were imposed alternately on each mouse. All combinations of the two corners out of four corners were presented twice each.

The number of trials, which is defined as the sum of the success and failure responses required to reach the upper criterion, was used to assess the efficiency of learning and behavioral flexibility at each task phase. In addition, the average numbers of nosepokes per active rewarding corner, inactive rewarding corner and nonrewarding corners were calculated as secondary indices of behavioral flexibility. This is based on the notion that, no matter what type of corner visit it is, more than a single nosepoke is redundant in this task—the second nosepoke will not provide any further reward in the active rewarding corner, and neither the inactive rewarding corner nor the nonrewarding corner provide reward, regardless of how many nosepoke attempts a mouse may make. Therefore, repetitive nosepokes in each corner visit can be interpreted as an inability to flexibly terminate the meaningless actions; in other words, behavioral inflexibility derived from impulsive or perseverative characteristics.

#### Behavioral inhibition and sustained attention test

This test was designed based on the protocol of an operant schedule called differential reinforcement of low rates with limited hold (DRL-LH): the protocol assesses whether mice could maintain impulsive nosepoking behavior under control for a certain amount of time and whether they could sustain attention during that time.

In this test, all mice were permitted to obtain water as a reward in any of the four corners by making nosepokes in a way that followed these rules: when a mouse entered a corner, the leftmost and rightmost LEDs above nosepoke holes were lit yellow. The LED lights turned off when the mouse made its first nosepoke on either side, but the door did not open. Then, after a certain amount of time (delay time), the LED lights turned back on (‘reward signal’), at which time the mouse could obtain a reward by making its second nosepoke. The door opened for 4,000 ms in response to the nosepoke; after it closed, the trial for that corner visit ended, meaning that the mouse had to leave the corner and re-enter it for more reward.

On the first day of the training phase (Training Day 1), the delay time was set to 0, so mice were rewarded for nosepoking twice in a row. On Training Day 2, the delay time was set to 100 ms, on Training Day 3 to 200 ms and on Training Days 4–5 to 500 ms. If the mouse nosepoked during the delay time, the timer was set back to zero, meaning that mice had to suppress nosepoke activity during the delay time to obtain rewards in an efficient manner.

At the end of Training Day 5, the following three test sessions were conducted (4 days per session): in Session 1, a delay time of 500 ms, 1,000 ms or 1,500 ms was presented randomly in each trial. If a nosepoke was made during the delay time, the timer was set back to zero and recorded as one premature response. In the analysis, the percent of premature responses during each delay time was used as an indicator of the difficulty of behavioral inhibitory control. A nosepoke during the reward signal opened the door for 4,000 ms to permit mice to drink water as a reward. However, the reward signals were presented only for 5 s, and if no nosepoke was made during that time, the reward opportunity was lost for that trial. The response time between the presentation of the reward signal and the nosepoke was used as a measure of sustained attention. In Session 2, a delay time of 1,000 ms, 1,500 ms or 2,000 ms was presented randomly and, in Session 3, a delay time of 1,500 ms, 2,000 ms or 2,500 ms was presented randomly, otherwise the rules were the same as for Session 1. For each of Sessions 1, 2 and 3, data from the second day onward were used to evaluate performance, as the first day of each 4-day session required familiarization with the new delay time condition.

#### Taste sensitivity and motivation/apathy

In the first part of this test, sensitivity to taste was evaluated using differently flavored water. After confirming that there was no significant difference in the general taste sensitivity between the groups, the motivation to obtain a highly preferred sweet reward was evaluated based on an operant schedule called progressive ratio^83^.

The following reagents were used for the taste sensitivity test: a 0.1% aqueous solution of saccharin (FUJIFILM Wako Pure Chemical Corporation, cat. no. 199-08665), a 3 mM aqueous solution of citric acid (FUJIFILM Wako Pure Chemical Corporation, cat. no. 038-06925) and a 0.1 mM aqueous solution of (−)-quinine sulfate dihydrate (FUJIFILM Wako Pure Chemical Corporation, cat. no. 175-00382). Saccharin was used at a concentration known from previous evaluations to be highly preferred by C57BL/6J strain mice. Citric acid and quinine, which induce no preference in the strain at any concentrations, were used at concentrations known to achieve a neutral to slight avoidance response in C57BL/6J strain mice.

In the taste sensitivity test and the subsequent motivation test, mice were able to drink flavored or plain water freely from any of the four corners; in two corners, they were able to drink flavored water from the right nosepoke holes and plain water from the left. In the remaining two corners, the positions of the left and right bottles were reversed.

The protocol for the taste sensitivity test was as follows. When a mouse visited a corner, the doors of the left and right nosepoke holes were opened automatically. When the mouse nosepoked on either side, the door on the side of the nosepoke closed after 1,500 ms (henceforth called the ‘tasting trial’). After the tasting trial, when the mouse nosepoked the closed door again, the door opened for 2,000 ms (hereafter called ‘preference-based choice’). The mouse could repeat the preference-based choice as many times as it wanted in a single corner visit. In the analysis, the percentage of preference-based choices made for flavored water out of the total choices made for both types of solutions was used as a taste sensitivity index. The sensitivity to each type of flavored water was assessed by testing over 2 days for each water type. Day 1 began at 12:00 noon, 4 h after the start of the light period, with the flavored and water bottles inserted into the IntelliCage. On the second day of the test, the positions of the bottles on the left and right sides of each corner were reversed at 12:00 noon. Data were collected until 12:00 noon the next day, when the study was completed for a particular flavor. The mean value of the preference-based choice rate for the flavored water on each day was used as a representative value for comparison between groups. Taste sensitivities were evaluated in the order of saccharin as a sweet taste, citric acid as a sour taste and quinine as a bitter taste.

After confirming that no significant difference in preference for the 0.1% saccharin solution was apparent between the groups, the degree of motivation for acquiring this sweet reward was tested using the progressive ratio operant schedule. In this test, as in the previous sensitivity test protocol, the mice were able to choose between the saccharin solution and plain water at each corner, but the following rules were added: for both saccharin water and plain water, preference-based choice was initially considered to have been made by making two nosepokes in a row after the tasting trial, where mice were given 4,000 ms to drink the water. Thereafter, each time the total number of preference-based choices exceeded 50, the number of nosepokes required to drink saccharin water was increased one by one until ten nosepokes were required for mice to obtain an opportunity to drink saccharin water. During this period, the rates of preference-based choice for saccharin water to total preference-based choice were used in the analysis as an index of motivation to seek palatable pleasure.

#### Social/competitive dominance in a group

In this test, mice were acclimated to a water supply restriction schedule required for the test by progressively limiting the water-available period during the day as follows (Adaptation Days 1–10): In Adaptation Days 1–2, mice were able to drink water only during the 12 h of the dark period (20:00–8:00); in Adaptation Days 3–4 from 21:00 to 05:00; in Days 5–6 from 22:00 to 04:00 and in Days 7–10 from 22:00 to 02:00. During this period, mice were allowed to visit any corner during the water-available period, and the door opened with each nosepoke, giving them 2,000 ms of drinking opportunity. They were able to drink water repeatedly during a single corner visit.

To allow mice to distinguish between the water-available period and the water not-available period, the following conditions were added. First, the water-available period was signaled by an alarm device (Tokyo Devices, cat. no. IWT120-USB) fitted to the outside of the IntelliCage (but visible from inside the cage). The device was run by a Windows batch file to beep for 20 s at the beginning of the period and a flashing red-colored LED light, which is known to be perceived as a low-intensity light for mice, until the end of the period. Also, when a mouse entered the corner during the water-available period, the leftmost and rightmost LED lights both lit up in red.

The test was conducted for 11 days starting from the day after Adaptation Day 10. Here, as in Adaptation Days 7–10, 4 h from 22:00 to 02:00 were set as the water-available period, and the following conditions were added: mice could open the door only once per corner visit; mice were not able to drink water by re-entering the same corner repeatedly. Under these conditions, it is known that mice in the IntelliCage behave competitively for the opportunity to access the limited number of water sources (four corners), at the beginning of the test, especially during the first 5 min, and that they behave dominantly or subordinately in this competitive situation depending on their growing environment, genetic factors and so on^84–86^.

In the analysis, we used the corner visit duration (corner occupancy) of 5 and 10 min immediately after the start of the test as an index of competitive behavior or dominance. The mean total corner visit duration during the daily water-available period was calculated to examine whether there was any difference in the basic motivation of the mice toward water intake.

### Statistics

When comparing means in three or more groups, statistical analysis was performed by one-way or two-way analysis of variance (ANOVA) (depending on the number of independent variables assessed) followed by a post hoc test; Tukey’s test was applied if the data passed the Brown–Forsythe equal variance tests. If the normality test or the equal variance test did not pass, Kruskal–Wallis one-way ANOVA on the ranks followed by Dunn’s multiple comparison tests was performed. A *χ*^2^ goodness of fit test was performed to investigate statistical difference for sample group (*MAPT* KI, *MAPT*^S305N;Int10+3^ KI and PSP) comparing the expected distribution (mean of observed values) of the samples from the observed values of each group. To identify outliers, we used the ROUT method provided by Prism software (San Diego). The specific statistical test performed in each assessment can be found in the respective figure legend and individual genotype comparison results can be found in the statistical summary. All data are shown as the mean ± s.e.m. All data were analyzed and displayed with Prism software. PCA analysis and plotting used Python (Python software foundation v.3.9.12, available at https://www.python.org/) and the scientific Python stack: scipy (v.1.8.1), numpy (v.1.23.1), scikit-learn (v.0.19.3) and matplotlib (v.3.5.2). No statistical methods were used to predetermine sample sizes but our sample sizes are similar to those reported in previous publications^2,27–30,32^. Animals were assigned randomly to different experimental groups. The experiments were blinded to the genotype of the animal. In the behavioral test with IntelliCage, the mice were implanted with RFID microchip allowing for recognition of each mouse; therefore, for this experiment we could not be blinded to the genotype. However, we randomly chose the mice across the genotype and transferred them to the IntelliCage apparatus. Experiments with human samples were performed blindly.

### Reporting summary

Further information on research design is available in the Nature Portfolio Reporting Summary linked to this article.

## Online content

Any methods, additional references, Nature Portfolio reporting summaries, source data, extended data, supplementary information, acknowledgements, peer review information; details of author contributions and competing interests; and statements of data and code availability are available at 10.1038/s41593-024-01829-7.

## Supplementary information

Reporting Summary

Supplementary Table 1 Summary of substitution efficiency and generation of tau mutants after BE microinjection targeting *MAPT*-P301 and *MAPT*-Int10+3.

Supplementary Table 2 Summary of off-target candidate sites predicted by COSMID for *MAPT*^Int10+3^ KI and *MAPT*^S305N;Int10+3^ KI mice.

Supplementary Table 3 Antibodies used for immunoblotting and immunohistochemical analyses.

Supplementary Table 4 Primer list for in vitro transcription and several PCR tests.

Supplementary Table 5 Human sample information.

## Source data

Source Data Statistical source data for Figs. 2 and 4–8, and Extended Data Figs. 3 and 6–10, statistics summary for Figs. 2 and 4–8, and Extended Data Figs. 3 and 6–10, and unprocessed western blot and gels for Fig. 3.

## Data Availability

The datasets generated during and/or analyzed during the current study are available from the corresponding author on request. The whole-genome resequencing data are deposited in SRA (NCBI) under the accession number SAMN43357596 (*MAPT* KI), SAMN43357597 (*MAPT*^Int10+3^ KI) and SAMN43357598 (*MAPT*^S305N;Int10+3^ KI) under project accession number PRJNA1152251. The mutant mice described will be shared with the research community upon request to T.C.S. and RIKEN BRC. Source data are provided with this paper.
