# Supplementary material for: In vivo hyperphosphorylation of tau is associated with synaptic loss and behavioral abnormalities in the absence of tau seeds
Source: Nat Neurosci. 2024 Dec 24;28(2):293–307. doi: 10.1038/s41593-024-01829-7 (PMC11802456; doi:10.1038/s41593-024-01829-7)
Supplement: Supplementary file 1 — Reporting Summary [file 41593_2024_1829_MOESM1_ESM.pdf]

nature portfolio

Corresponding author(s): Naoto Watamura
Martha S. Foiani
Karen E. Duff
Takaomi C. Saido

Last updated by author(s): Aug 20, 2024

Reporting Summary

Nature Portfolio wishes to improve the reproducibility of the work that we publish. This form provides structure for consistency and transparency in reporting. For further information on Nature Portfolio policies, see our Editorial Policies and the Editorial Policy Checklist.

Statistics

For all statistical analyses, confirm that the following items are present in the figure legend, table legend, main text, or Methods section.

n/a	Confirmed	
☐	☒	The exact sample size (*n*) for each experimental group/condition, given as a discrete number and unit of measurement
☐	☒	A statement on whether measurements were taken from distinct samples or whether the same sample was measured repeatedly
☐	☒	The statistical test(s) used AND whether they are one- or two-sided<br>*Only common tests should be described solely by name; describe more complex techniques in the Methods section.*
☐	☒	A description of all covariates tested
☐	☒	A description of any assumptions or corrections, such as tests of normality and adjustment for multiple comparisons
☐	☒	A full description of the statistical parameters including central tendency (e.g. means) or other basic estimates (e.g. regression coefficient) AND variation (e.g. standard deviation) or associated estimates of uncertainty (e.g. confidence intervals)
☐	☒	For null hypothesis testing, the test statistic (e.g. *F*, *t*, *r*) with confidence intervals, effect sizes, degrees of freedom and *P* value noted<br>*Give P values as exact values whenever suitable.*
☒	☐	For Bayesian analysis, information on the choice of priors and Markov chain Monte Carlo settings
☒	☐	For hierarchical and complex designs, identification of the appropriate level for tests and full reporting of outcomes
☒	☐	Estimates of effect sizes (e.g. Cohen's *d*, Pearson's *r*), indicating how they were calculated

Our web collection on statistics for biologists contains articles on many of the points above.

Software and code

Policy information about availability of computer code

Data collection	LC-MS/MS: Dionex 3000 LC-system (Thermo Fisher Scientific). Immunohistochemistry: Zen Black 3.0 (Zeiss) and NanoZoomer Digital Pathology C9600 (Hamamatsu Photonics). Biosensor cells: LSRFortessa Flow Cytometer (BD Biosciences) and Opera Phenix Plus (Perkin Elmer). Classical Behavioral analysis (Y-maze, Open-field, Novel object location and Barnes-maze tests): O'hara & CO.LTD. IntelliCage: the software provided by the Phenovance Research & Technology,LLC.
Data analysis	Data representation: Prism 9.4.1 (GraphPad Software, Inc)<br>Image Analysis: Image J (1.53a) and Fiji software 1.0 (NIH), Imaris Cell Imaging Software 9.8 (Bitplane), and Definiences Tissue Studio (3.60).<br>Whole genome sequencing: Isaac Genome Alignment software (version 01.15.02.08) and Isaac Variant Caller (ICV) (version 2.0.13) Flow Cytometry: FCS express v7 (De Novo Software)<br>LC-MS/MS: Mascot Deamon v2.6/Mascot Distiller v2.6.3 (Matrix Science) and Skyline v20.1.0.31 (MacCoss lab)<br>Python (v.3.9.12) and the scientific python stack: scipy (v.1.8.1), numpy (v.1.23.1), scikit-learn (v.0.19.3), and matplotlib (v.3.5.2)

For manuscripts utilizing custom algorithms or software that are central to the research but not yet described in published literature, software must be made available to editors and reviewers. We strongly encourage code deposition in a community repository (e.g. GitHub). See the Nature Portfolio guidelines for submitting code & software for further information.

Data

Policy information about availability of data

All manuscripts must include a data availability statement. This statement should provide the following information, where applicable:

- Accession codes, unique identifiers, or web links for publicly available datasets
- A description of any restrictions on data availability
- For clinical datasets or third party data, please ensure that the statement adheres to our policy

The datasets generated during and/or analyzed during the current study are available from the source data or the corresponding author on request. The whole genome resequencing data are deposited in SRA (NCBI) under the accession number SAMN43357596 (MAPT KI), SAMN43357597 (MAPTInt10+3KI), and SAMN43357597 (MAPTS305N; Int10+3KI) under Project accession number PRJNA1152251.

Human research participants

Policy information about studies involving human research participants and Sex and Gender in Research.

Reporting on sex and gender	Information regarding sex and gender is included in Supplementary Table 5, however sex and gender were not taken into consideration when including patient samples, but availability of the tissues.
Population characteristics	Post-mortem samples of 3 AD patients (0 male, 3 female; age at death between 61-86y), 3 FTLD-IVS10+3 patients (3 male, 0 female; age at death between 42-57y), 1 FTLD-S305N patient (1 male, 0 female; age at death 46y), and 3 PSP patient (2 male, 1 female; age at death between 67-76) were analyzed. See Supplementary Table 5 for PMI, ApoE, Braak Stage,and brain region.
Recruitment	Samples were selected based on neuropathological examination and availability of the tissues, which is unlikely to have impacted the results and conclusions.
Ethics oversight	All brain samples were donated with informed consent in compliance with the 1998 data protection act and summarized in Supplemental Table 5. Ethical approval for the study was acquired from NHS and UCL ethics committee (QSBB UCLMTA06-23) and in accordance with the human tissue authority's code of practice and standards under license number UCLMTA 06-23, with an approved material transfer agreement.

Note that full information on the approval of the study protocol must also be provided in the manuscript.

Field-specific reporting

Please select the one below that is the best fit for your research. If you are not sure, read the appropriate sections before making your selection.

☒ Life sciences ☐ Behavioural & social sciences ☐ Ecological, evolutionary & environmental sciences

For a reference copy of the document with all sections, see nature.com/documents/nr-reporting-summary-flat.pdf

Life sciences study design

All studies must disclose on these points even when the disclosure is negative.

Sample size	The sample size used in this study are appropriate. FTLD-S305N patient samples were limited. No statistical methods were used to pre-determine sample sizes but our sample sizes are similar to those reported in previous publications (Watamura et al., 2022; Balan et al., 2021; Oizumi et al., 2020; Endo et al., 2012; Ishii et al., 2015; Hashimoto et al., 2019).
Data exclusions	In the behavioral test with IntelliCage, some mice were excluded because they died during test (Figure8, Supplemental Figure 9 and 10). In terms of other experiments, there is no data exclusions except for the outliers identified by ROUT method of Prism software in this study.
Replication	All presented data are representative of the same experiment performed in at least 3 animals. All experiments were replicated in at least two independent experiments unless stated otherwise.
Randomization	Animals were randomly assigned to different experimental groups. The allocation of human samples was random.
Blinding	The experimentalist were blinded to the genotype of the animal. In the behavioral test with IntelliCage, the mice were implanted with a small, glass-covered radio-frequency identification (RFID) microchip to recognize each mouse therefore the experimentalist were not blinded to the genotype. However, we randomly chose the mice across the genotype and transferred them to the IntelliCage apparatuses. Experiments with human samples were performed in a blinded fashion.

Reporting for specific materials, systems and methods

We require information from authors about some types of materials, experimental systems and methods used in many studies. Here, indicate whether each material, system or method listed is relevant to your study. If you are not sure if a list item applies to your research, read the appropriate section before selecting a response.

Materials & experimental systems

n/a	Involved in the study
☐	☒ Antibodies
☐	☒ Eukaryotic cell lines
☒	☐ Palaeontology and archaeology
☐	☒ Animals and other organisms
☒	☐ Clinical data
☒	☐ Dual use research of concern

Methods

n/a	Involved in the study
☒	☐ ChIP-seq
☐	☒ Flow cytometry
☒	☐ MRI-based neuroimaging

Antibodies

Antibodies used

CP-13 Kindly provided by Cristina D'Abramo WB1:500 IHC1:500
AT8 Inoggenetics #90206 (anti-PHF-TAU)/Thermo Fisher MN1020b WB1:2000 IHC1:500
PHF-1 Kindly provided by Peter Davis WB1:2000 IHC1:1000
AT180 Thermo #MN-1040 IHC1:100
AT270 Thermo #1050 IHC1:100
TOC1 Kindly provided by Nicholas Kanaan IHC1:1500
T22 Merck ABN454 IHC1:500
MC1 Kindly provided by Cristina D'Abramo IHC1:500
Tau13 Santa Cruz #sc-21796 WB1:2000
Tau5 Thermo #AHB0042 WB1:2000
HT7 Thermo #MN-1000 WB1:2000
K9JA Dako # A0024 WB1:10000
RD3 Merk Millipore #05-803 IHC1:100
RD4 Merk Millipore #05-804 IHC1:100
Synaptotagmin Synaptic System 105-002 IHC1:500
Homer Synaptic System 160-004 WB1:1000 IHC1:500
VGLUT1 Synaptic System 135-303 IHC1:500
β-actin Sigma A5441 WB1:5000
Synaptophysin ab14692 WB1:1000
PSD-95 ab18258 WB 1:1000
Tubulin Sigma MAB1864 WB 1:2000

Secondary antiodies
Goat anti-Rabbit IgG Secondary antibody, Alexa Fluor TM 488 Invitrogen #A-11008 IHC1:500
Goat anti-Chicken IgY Secondary antibody, Alexa Fluor TM 568 Invitrogen #A-11041 IHC1:500

Validation

CP-13, AT8, PHF-1, Tau13, Tau5, HT7 and K9JA were validated with Tau KO mice for WB. Validation studies for other antibodies can be found on the manufacture's website.

Eukaryotic cell lines

Policy information about cell lines and Sex and Gender in Research

Cell line source(s)

ATCC

Authentication

HEK Tau RD P301S FRET Biosensor was obtained from ATCC (CRL-3275) .
HEK Tau RD S305N-YFP was created in house.

Mycoplasma contamination

HEK Tau RD P301S FRET Biosensor was not manipulated in our laboratory. HEK Tau RD S305N-YFP was not tested for the mycoplasma contamination.

Commonly misidentified lines
(See ICLAC register)

Name any commonly misidentified cell lines used in the study and provide a rationale for their use.

Animals and other research organisms

Policy information about studies involving animals; ARRIVE guidelines recommended for reporting animal research, and Sex and Gender in Research

Laboratory animals	Animals used in this study: C57BL/6J (WT) Stock no. 000664, MAPT KI mice (Saito et al., 2019 and Hashimoto et al., 2019). MAPTInt10 +3 KI, and MAPTS305N; Int10+3 KI mice were generated by the base editors based on the MAPT KI mice (Please see methods). PS19 mice (Yoshiyama et al., 2007) were used as positive controls. ICR (Jcl) mice were used as zygote donors and foster mothers. Animals between the ages of 3-30 months were used in this study.
Wild animals	No wild animals were used in this study
Reporting on sex	Both female and male mice were used in this study
Field-collected samples	No field collected samples were used in this study
Ethics oversight	All animal experiments were conducted in accordance with guidelines of the RIKEN Center for Brain Science (W2021-2-020(3)) and UK animal act, 1968 (PP7490525), , and following local ethics committee approval

Note that full information on the approval of the study protocol must also be provided in the manuscript.

Flow Cytometry

Plots

Confirm that:

- ☒ The axis labels state the marker and fluorochrome used (e.g. CD4-FITC).
- ☒ The axis scales are clearly visible. Include numbers along axes only for bottom left plot of group (a 'group' is an analysis of identical markers).
- ☒ All plots are contour plots with outliers or pseudocolor plots.
- ☒ A numerical value for number of cells or percentage (with statistics) is provided.

Methodology

Sample preparation	Tau RD P301S FRET biosensor cells were harvested with 0.25% trypsin and fixed in 4% PFA for 10 min, then resuspended in flow cytometry buffer (1X PBS and 1mM EDTA). An LSRFortessa Flow Cytometer (BD Biosciences) was used to perform FRET flow cytometry on the Tau RD P301S FRET biosensors. Briefly, single cells that were double-positive for YFP and CFP were identified and FRET-positive cells within this population were quantified. The percentage of FRET (the number of FRET-positive cells per total cell count) and the Integrated FRET density (the product of percent positivity and median fluorescence intensity) were used as output measures. Data analysis was performed using FCS express v7 (De Novo Software) and GraphPad Prism v9.4.1 for Mac OS X.
Instrument	An LSRFortessa Flow Cytometer (BD Biosciences)
Software	FCS express v7 (De Novo Software) and GraphPad Prism v9.4.1 for Mac OS X.
Cell population abundance	FRET was quantified as previously described (Furman et al., 2015).
Gating strategy	Single cells that were double-positive for YFP and CFP were identified and FRET-positive cells within this population were quantified. The percentage of FRET (the number of FRET-positive cells per total cell count) and the Integrated FRET density (the product of percent positivity and median fluorescence intensity) were used as output measures. Gating strategy was followed with previous literature (Furman et al., 2015) and described in the Extended Data Fig. 6b.

☒ Tick this box to confirm that a figure exemplifying the gating strategy is provided in the Supplementary Information.

March 2021
