# Supplementary material for: In vivo hyperphosphorylation of tau is associated with synaptic loss and behavioral abnormalities in the absence of tau seeds
Source: Nat Neurosci. 2024 Dec 24;28(2):293–307. doi: 10.1038/s41593-024-01829-7 (PMC11802456; doi:10.1038/s41593-024-01829-7)
Supplement: Supplementary file 2 — Summary of substitution efficiency and generation of tau mutants after BE microinjection targeting MAPT-P301 and MAPT-Int10+3. [file 41593_2024_1829_MOESM2_ESM.pdf]

Mutations	Substitution Efficiency (%)
No substitution	7/166 (4.2)
Indel	10/166 (6.0)
P301L	79/166 (47.6)
P301S	19/166 (11.4)
P301V	1/166 (0.6)
Int10+3 G>A	36/166 (21.7)
P301L; Int10+3 G>A	9/166 (5.4)
P301S; Int10+3 G>A	4/166 (2.4)
S305N; Int10+3 G>A	1/166 (0.6)
