# Supplementary material for: In vivo hyperphosphorylation of tau is associated with synaptic loss and behavioral abnormalities in the absence of tau seeds
Source: Nat Neurosci. 2024 Dec 24;28(2):293–307. doi: 10.1038/s41593-024-01829-7 (PMC11802456; doi:10.1038/s41593-024-01829-7)
Supplement: Supplementary file 3 — Summary of off-target candidate sites predicted by COSMID for MAPTInt10+3 KI and MAPTS305N;Int10+3 KI mice. [file 41593_2024_1829_MOESM3_ESM.pdf]

Off-target candidate	Query type	Mismatch	Chr Position	Gene	Annotation	Off-target *MAPT* KI$^{Inton10+3\ G>A}$	Off-target *MAPT* KI$^{S305N;\ Inton10+3\ G>A}$
MAPT-P301-1	No indel	3	Chr11:70351826-70351849	*Alox15*	Intron	-	-
MAPT-P301-2	No indel	3	Chr4:119200276-119200299	*Svbp*	Intron	○	○
MAPT-P301-3	No indel	3	Chr9:64060473-64060496		Intergenic	-	-
MAPT-P301-4	No indel	3	Chr6:95725509-95725532		Intergenic	-	-
MAPT-P301-5	No indel	3	Chr12:64966002-64966025	*Togaram1*	Exon	○	○
MAPT-P301-6	No indel	3	Chr14:78141609-78141632		Intergenic	-	-
MAPT-P301-7	No indel	3	Chr2:126701111-126701134		Intergenic	-	-
MAPT-P301-8	No indel	3	Chr12:72964402-72964425		Intergenic	-	-
MAPT-P301-9	No indel	3	Chr1:180813179-180813202	*H3f3a*	Intron	-	-
MAPT-P301-10	No indel	3	Chr6:146888630-146888653	*Ppfibp1*	Non-coding	-	-
MAPT-P301-11	Del 8	2	Chr9:121021200-121021222	*Ulk4*	Intron	-	-
MAPT-P301-12	Del 3	2	ChrX:87776462-87776484	*Il1rapl1*	Intron	-	-
MAPT-P301-13	Del 2	2	Chr16:18687059-18687081		Intergenic	-	-
MAPT-P301-14	Del PAM 2	2	Chr2:167736542-167736564		Intergenic	-	-
MAPT-P301-15	Ins 17	2	Chr10:118675703-118675727		Intergenic	-	-
MAPT-P301-16	Ins 16	2	Chr2:133528883-133528907		Intergenic	-	-
MAPT-P301-17	Ins 13	2	Chr14:26429735-26429759	*Slmap*	Intron	○	-
MAPT-P301-18	Ins 7	2	Chr6:146527101-146527125		Intergenic	-	-
MAPT-P301-19	Ins 5	2	Chr2:131262710-131262734	*Pank2*	Intron	-	-
MAPT-intron10+3 G>A-1	No indel	3	Chr14:31275639-31275661	*Dnah1*	Intron	-	-
MAPT-intron10+3 G>A-2	No indel	3	Chr10:67285234-67285256	*Nrbf2*	Non-coding	-	-
MAPT-intron10+3 G>A-3	No indel	3	Chr3:108148064-108148086		Intergenic	-	-
MAPT-intron10+3 G>A-4	No indel	3	Chr5:137062275-137062297	*Serpine1*	Non-coding	-	-
MAPT-intron10+3 G>A-5	No indel	3	Chr6:72577777-72577799	*Elmod3*	Intron	-	-
MAPT-intron10+3 G>A-6	No indel	3	Chr5:64508703-64508725		Intergenic	-	-
MAPT-intron10+3 G>A-7	Del 18	2	Chr6:59413774-59413795	*Gprin3*	Intron	-	-
MAPT-intron10+3 G>A-8	Del 18	2	Chr7:96207591-96207612	*Tenm4*	Intron	-	-
MAPT-intron10+3 G>A-9	Del 17	2	Chr2:168700314-168700335	*Atp9a*	Intron	-	-
MAPT-intron10+3 G>A-10	Del 16	2	Chr2:146621362-146621383		Intergenic	-	-
MAPT-intron10+3 G>A-11	Del 16	2	Chr8:69915135-69915156	*Gatad2a*	Intron	-	-
MAPT-intron10+3 G>A-12	Del 16	2	Chr11:99056068-99056089		Intergenic	-	-
MAPT-intron10+3 G>A-13	Del 15	2	Chr2:167736538-167736559		Intergenic	-	-
MAPT-intron10+3 G>A-14	Del 14	2	Chr1:82288807-82288828	*Irs1*	Exon	-	-
MAPT-intron10+3 G>A-15	Del 14	2	Chr4:150626514-150626535		Intergenic	-	-
MAPT-intron10+3 G>A-16	Del 14	2	Chr17:30516288-30516309	*Btbd9*	Intron	-	-
MAPT-intron10+3 G>A-17	Del 14	2	Chr8:112093736-112093757		Intergenic	-	-
MAPT-intron10+3 G>A-18	Del 14	2	Chr12:84648519-84648540	*Vrtn*	Exon	-	-
MAPT-intron10+3 G>A-19	Del 14	2	Chr11:87664708-87664729	*Rnf43*	Exon	-	-
MAPT-intron10+3 G>A-20	Del 13	2	Chr4:139161305-139161326	*Gm33304*	Non-coding	-	-
MAPT-intron10+3 G>A-21	Del 12	2	Chr18:80778335-80778356	*Atp9b*	Intron	-	-
MAPT-intron10+3 G>A-22	Del 12	2	Chr2:119770882-119770903	*Rpap1*	Intron	-	-
MAPT-intron10+3 G>A-23	Del 12	2	Chr6:109857978-109857999		Intergenic	-	-
MAPT-intron10+3 G>A-24	Del 12	2	Chr5:142468117-142468138	*Ap5z1*	Exon	-	-
MAPT-intron10+3 G>A-25	Del 11	2	Chr17:25386575-25386596	*Cacna1h*	Intron	-	-
MAPT-intron10+3 G>A-26	Del 11	2	Chr9:119555524-119555545	*Scn5a*	Intron	-	-
MAPT-intron10+3 G>A-27	Del 9	2	Chr9:43536132-43536153		Intergenic	-	-
MAPT-intron10+3 G>A-28	Del 9	2	Chr7:125974764-125974785	*Gsg1l*	Intron	-	-
MAPT-intron10+3 G>A-29	Del 6	2	Chr12:91965237-91965258		Intergenic	-	-
MAPT-intron10+3 G>A-30	Del 4, or Del 5	2	Chr11:118367373-118367394		Intergenic	-	-
MAPT-intron10+3 G>A-31	Del 4, or Del 5	2	Chr4:43394834-43394855	*Rusc2*	Intron	-	-
MAPT-intron10+3 G>A-32	Del 4, or Del 5	2	Chr5:121818513-121818534	*Sh2b3*	Exon	-	-
MAPT-intron10+3 G>A-33	Del 3	2	Chr12:21295846-21295867	*Cpsf3*	Intron	-	-
MAPT-intron10+3 G>A-34	Del 3	2	Chr9:113870747-113870768	*Clasp2*	Intron	-	-
MAPT-intron10+3 G>A-35	Del 3	2	Chr6:125633288-125633309	*Vwf*	Intron	-	-
MAPT-intron10+3 G>A-36	Del 3	2	Chr2:130840193-130840214	*930402H24R*	Non-coding	-	-
MAPT-intron10+3 G>A-37	Del 1, or Del 2	2	Chr17:49049099-49049120	*Lrfn2*	Intron	-	-
MAPT-intron10+3 G>A-38	Ins 17	2	Chr16:22558979-22559002	*Dgkg*	Intron	-	-
MAPT-intron10+3 G>A-39	Ins 16	2	Chr15:74631843-74631866	*Mroh4*	Intron	-	-
MAPT-intron10+3 G>A-40	Ins 15	2	Chr17:54077434-54077457		Intergenic	-	-
MAPT-intron10+3 G>A-41	Ins 13	2	Chr7:139648287-139648310	*Cfap46*	Intron	-	-
MAPT-intron10+3 G>A-42	Ins 13	2	Chr2:30194586-30194609	*Kyat1*	Intron	-	-
MAPT-intron10+3 G>A-43	Ins 6	2	Chr8:71290281-71290304	*Myo9b*	Non-coding	-	-
MAPT-intron10+3 G>A-44	Ins 6	2	Chr5:138859529-138859552		Intergenic	-	-
MAPT-intron10+3 G>A-45	Ins 6	2	Chr16:93903368-93903391	*Chaf1b*	Intron	-	-
MAPT-intron10+3 G>A-46	Ins 1	2	Chr13:63111073-63111096	*Aopep*	Intron	-	-
