# Supplementary material for: In vivo hyperphosphorylation of tau is associated with synaptic loss and behavioral abnormalities in the absence of tau seeds
Source: Nat Neurosci. 2024 Dec 24;28(2):293–307. doi: 10.1038/s41593-024-01829-7 (PMC11802456; doi:10.1038/s41593-024-01829-7)
Supplement: Supplementary file 4 — Antibodies used for immunoblotting and immunohistochemical analyses. [file 41593_2024_1829_MOESM4_ESM.pdf]

The following antibodies were used at the dilutions described below.

Antibody		Dilution	
		WB	IHC
CP-13	Kindly provided by Cristina D'Abramo	1:500	1:500
AT8	Inoggenetics #90206 (anti-PHF-TAU) and ThermoFisher MN1020b	1:2000	1:500
PHF-1	Kindly provided by Cristina D'Abramo	1:2000	1:1000
AT180	Thermo #MN-1040	-	1:100
AT270	Thermo #1050	-	1:100
TOC1	Kindly provided by Lester I Binder	-	1:1500
T22	Merck #ABN454		1:500
MC1	Kindly provided by Peter Davis	-	1:500
Tau13	Santa Cruz #sc-21796	1:2000	-
Tau5	Thermo #AHB0042	1:2000	-
HT7	Thermo #MN-1000	1:2000	-
K9JA	Dako # A0024	1:10000	-
RD3	Merk Millipore #05-803	-	1:100
RD4	Merk Millipore #05-804	-	1:100
Synaptotagmin	Synaptic System 105-002	-	1:500
Homer1	Synaptic System 160-004	1:1000	1:500
VGLUT1	Synaptic System 135-303	-	1:500
β-actin	Sigma A5441	1:5000	-
Synaptophysin	abcum ab14692	1:1000	-
PSD-95	abcum ab18258	1:1000	-
Tubulin	Sigma MAB1864	1:2000	-
Secondary antiodies			
Goat anti-Rabbit IgG Secondary antibody, Alexa Fluor ™ 488	Invitrogen #A-11008		1:500
Goat anti-Chicken IgY Secondary antibody, Alexa Fluor ™ 568	Invitrogen #A-11041		1:500
