# Supplementary material for: In vivo hyperphosphorylation of tau is associated with synaptic loss and behavioral abnormalities in the absence of tau seeds
Source: Nat Neurosci. 2024 Dec 24;28(2):293–307. doi: 10.1038/s41593-024-01829-7 (PMC11802456; doi:10.1038/s41593-024-01829-7)
Supplement: Supplementary file 5 — Primer list for in vitro transcription and several PCR tests. [file 41593_2024_1829_MOESM5_ESM.pdf]

***In vitro* transcription for BE3**	
T7-BE3-F	GCCGCTAATACGACTCACTATAG
T7-BE3-R	GCGGGTTTAAACTCAATGGT
***In vitro* transcription for sgRNAs**	
sgRNA-MAPT-P301	TAATACGACTCACTATAGGTCCCGGGAGGCGGCAGTGGTTTTAGAGCTAGAA
sgRNA-MAPT-Intron10+3 G>A	TAATACGACTCACTATAGGACTCACACTGCCGCCTCCGTTTTAGAGCTAGAA
CRISPRscan tail primer	AAAAGCACCGACTCGGTGCCACTTTTTCAAGTTGATAACGGACTAGCCTTATTTTAACTTGCTATTTCTAGCTCTAAAAC
**Real-time PCR for tau**	
3R-Tau F	GTCCGTACTCCACCCAAGTC
3R-Tau R	TTTGTAGACTATTTGCACCTTCC
4R-Tau F	GAAGCTGGATCTTAGCAACG
4R-Tau R	GACGTGTTTGATATTATCCT
Total Tau F	AGCCAAGACATCCACACGTT
Total Tau R	ATCAGAGGGTCTGAGCTACCA
G3PDH F	CCATGGCACCGTCAAGGCTGA
G3PDH R	GCCAGTAGAGGCAGGGATGAT
**RT PCR for tau**	
Tau F	AAGTCGCCGTCTTCCGCCAAG
Tau R	GTCCAGGGACCCAATCTTCGA
**Sangar-sequencing for off-target sites**	
Svbp F	GGGGGTCAGAGTGTTTTCAA
Svbp R	GGAGCTGGGGAGCTAAAAAG
Togaram1 F	AATGGTTCCCTCCAGCTCTC
Togaram1 R	TTTTTCTCGCCTCTCATGGT
Slmap F	CACTGGTTGTTGGAGCATCT
Slmap R	GCCAAACAAGACATTCAGCA
Nploc4 F	CAAAATGACCATCGGGAGAG
Nploc4 R	AGTCATCCTGCTGGCTCAAC
