# Supplementary material for: In vivo hyperphosphorylation of tau is associated with synaptic loss and behavioral abnormalities in the absence of tau seeds
Source: Nat Neurosci. 2024 Dec 24;28(2):293–307. doi: 10.1038/s41593-024-01829-7 (PMC11802456; doi:10.1038/s41593-024-01829-7)
Supplement: Supplementary file 6 — Human sample information. [file 41593_2024_1829_MOESM6_ESM.pdf]

Sample	Gender	Age	PMI	ApoE genotype	Braak Tau Stage	Region	Import source	Number of cases
AD	F	61-86	36.55-94.05	N/A	6	Frontal Cortex	from QSBB	3
FTLD-Intron10+3	M	41.9-57	N/A	33,34	0	Frontal Cortex	from Mayo Clinic	3
FTLD-S305N	M	46	N/A	N/A	N/A	Frontal Cortex	from NHO Iou National Hospital	1
PSP	M/F	67-76	4-6	N/A	1-2	Motor Cortex	from Mayo Clinic	3
