# Supplementary figures and images for: In vivo hyperphosphorylation of tau is associated with synaptic loss and behavioral abnormalities in the absence of tau seeds
Source: Nat Neurosci. 2024 Dec 24;28(2):293–307. doi: 10.1038/s41593-024-01829-7 (PMC11802456; doi:10.1038/s41593-024-01829-7)

Fig.2 b

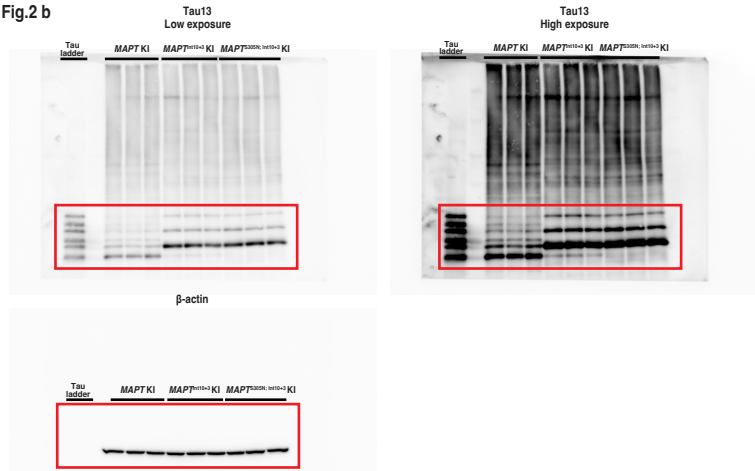

Fig.4 b

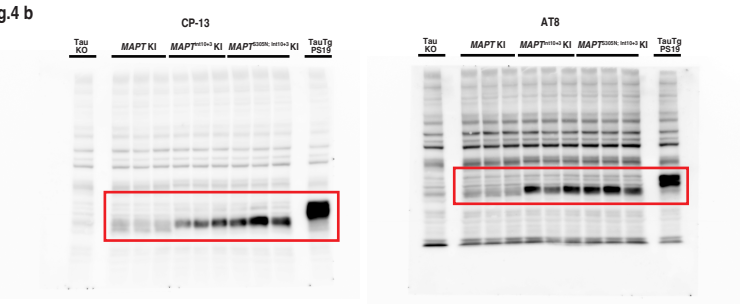

Fig.4 b

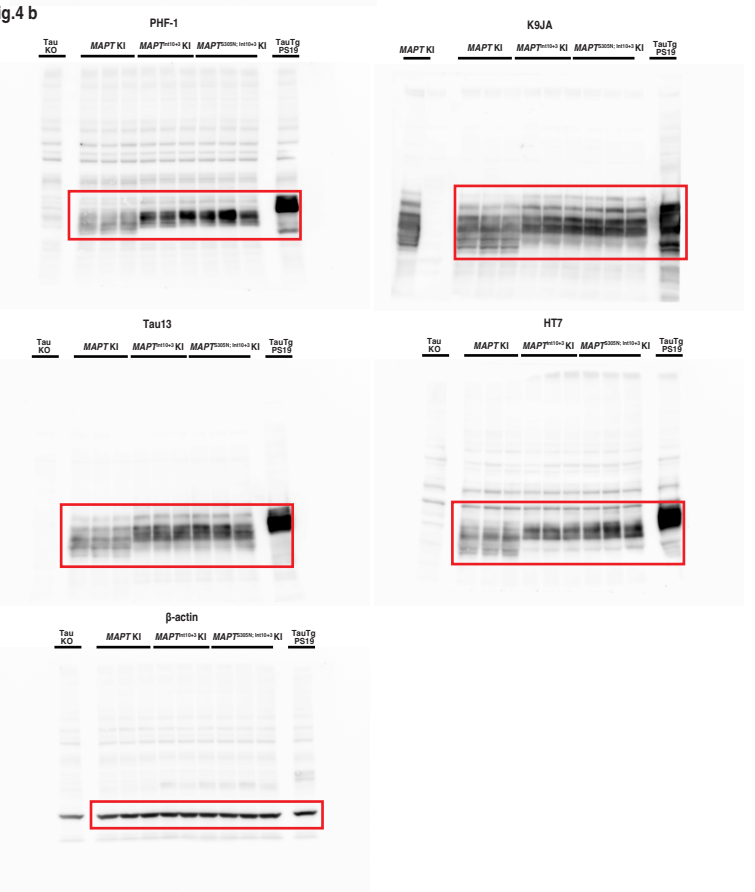

Fig.5 c

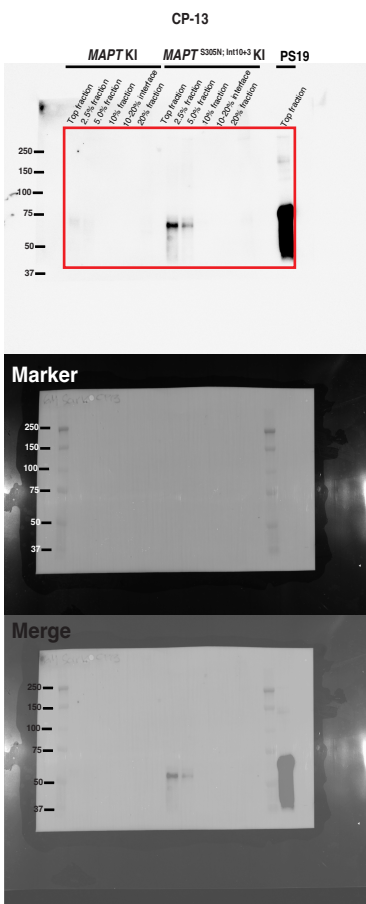

Fig.5 d

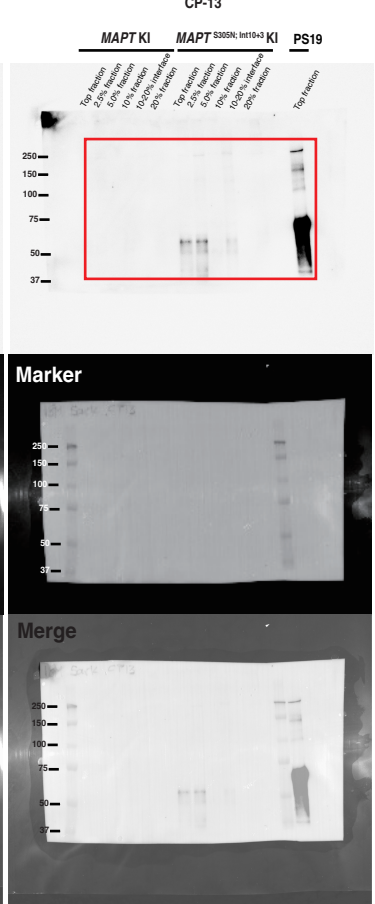

Extended Data Fig.3 a

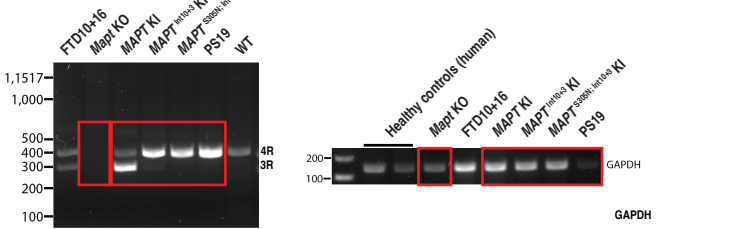

Extended Data Fig.7 c

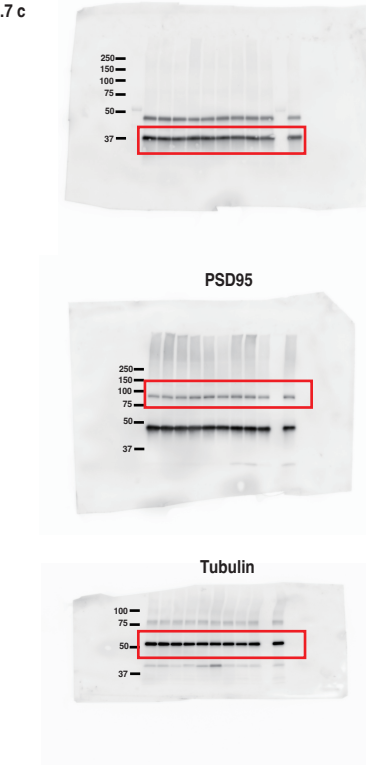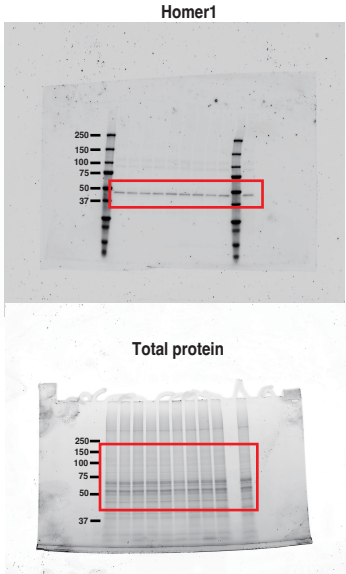

Extended Data Fig Fig.6 a

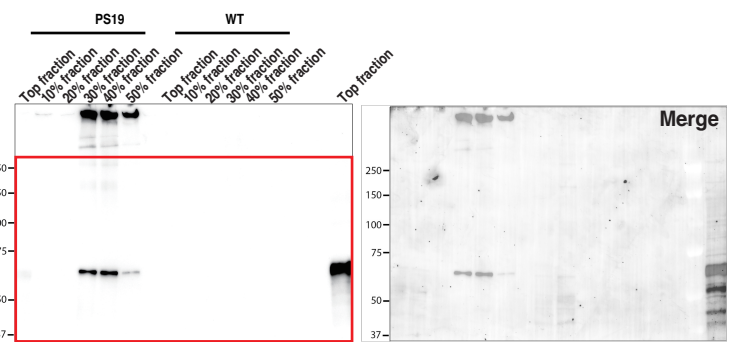

Supplement: Supplementary file 7 — Statistical source data for Figs. 2 and 4–8, and Extended Data Figs. 3 and 6–10, statistics summary for Figs. 2 and 4–8, and Extended Data Figs. 3 and 6–10, and unprocessed western blot and gels for Fig. 3. [file 41593_2024_1829_MOESM7_ESM.zip › Source_Data_Fig_3.pdf]
